# Identification of a strong and specific antichlamydial N-acylhydrazone

**DOI:** 10.1371/journal.pone.0185783

**Published:** 2017-10-03

**Authors:** Huirong Zhang, Anuj Kunadia, Yingfu Lin, Joseph D. Fondell, Daniel Seidel, Huizhou Fan

**Affiliations:** 1 Department of Pharmacology, Robert Wood Johnson Medical School, Rutgers, The State University of New Jersey, Piscataway, New Jersey, United States of America; 2 Department of Chemistry and Chemical Biology, School of Arts and Sciences, Rutgers, The State University of New Jersey, Piscataway, New Jersey, United States of America; University of California, San Francisco, UNITED STATES

## Abstract

Sexually transmitted *Chlamydia trachomatis* is an extremely common infection and often leads to serious complications including infertility and pelvic inflammatory syndrome. Several broad-spectrum antibiotics are currently used to treat *C*. *trachomatis*. Although effective, they also kill beneficial vaginal lactobacilli. Two N-acylhydrazones, CF0001 and CF0002, have been shown previously to inhibit chlamydial growth without toxicity to human cells and *Lactobacillus* spp. Of particular significance, the rate of random mutation leading to resistance of these inhibitors appears to be extremely low. Here, we report three analogs of CF0001 and CF0002 with significantly stronger inhibitory effects on chlamydiae. Even though the new compounds (termed SF1, SF2 and SF3) displayed slightly decreased inhibition efficiencies for a rare *Chlamydia* variant selected for CF0001 resistance (*Chlamydia muridarum* MCR), they completely overcame the resistance when used at concentrations of 75–100 μM. Importantly, SF1, SF2 and SF3 did not shown any toxic effect on lactobacilli, whereas SF3 was also well tolerated by human host cells. An effort to isolate SF3-resistant variants was unsuccessful. By comparison, variants resistant to rifampin or spectinomycin were obtained from smaller numbers of chlamydiae. Our findings suggest that SF3 utilizes an antichlamydial mechanism similar to that of CF0001 and CF0002, and will be more difficult for chlamydiae to develop resistance to, potentially making it a more effective antichlamydial agent.

## Introduction

Chlamydiae are Gram-negative bacteria replicating only inside eukaryotic host cells [1]. Of the more than 10 *Chlamydia* species, *C*. *pneumoniae* and *C*. *trachomatis* are important human pathogens. *C*. *pneumoniae* is an etiologic agent of pneumonia and bronchitis, and a possible risk factor for atherosclerosis [2] and late-onset Alzheimer disease [3, 4]. Worldwide, *C*. *trachomatis* is the most prevalent sexually transmitted bacterial pathogen [5, 6]. In the US, the number of people with sexually transmitted *C*. *trachomatis* infection consistently accounted for over 60% of the total number of cases of infection by some 60 different pathogens reported to the Centers for Disease Control and Prevention (CDC) in recent years [7, 8]. Yet, the CDC estimates the number of reported cases of *C*. *trachomatis* infection to be only one tenth of the actual number of infected people [9]. Some *C*. *trachomatis* serotypes cause conjunctivitis, and are the most common infectious microbe associated with blindness in various developing countries [10, 11]. Among the non-human-pathogenic chlamydiae, several are known zoonotic pathogens [12], whereas *C*. *muridarum* is used broadly to model human chlamydial infections in mice [13–15].

Although *C*. *trachomatis* is susceptible to several broad-spectrum antibiotics such as azithromycin and tetracyclines, most infected women do not seek medical treatment because they are either completely asymptomatic or only mildly symptomatic [16]. On the one hand, without proper antibiotic treatment, one-third of infected women can develop severe complications, including tubal factor infertility, pelvic inflammatory disease and ectopic pregnancy; on the other hand, treatment with broad spectrum antibiotics may lead to vaginal and gut dysbiosis [17–19]. Therefore, it is very desirable to develop antibacterials that narrowly target *Chlamydia*. Specific antichlamydials would also help reduce the risk of other bacterial pathogens developing resistance to common antibacterials.

Previous studies identified two N-acylhydrazones, CF0001 and CF0002, as specific antichlamydials [20]. While inhibiting all three *Chlamydia* species tested, *C*. *trachomatis*, *C*. *pneumoniae* and *C*. *muridarum*, CF0001 and CF0002 have no detectable toxicity to either host cells or vaginal lactobacilli. Another strikingly attractive feature of these two compounds is that it appears to be extremely difficult for chlamydiae to develop resistance to them. Accordingly, although a lengthy three month selection with stepwise increase in the CF0001 concentration led to isolation of a partially resistant variant, numerous repeated efforts failed to isolate additional resistant variants from *C*. *trachomatis* and *C*. *muridarum* even when mutagenized stocks were used as starting materials [20]. The high target selectivity of CF0001 and CF0002, combined with extremely low rates of resistance in chlamydiae, inspired us to develop more potent analogs. Here, we report three compounds that display increased antichlamydial activities while remaining nontoxic to vaginal lactobacilli. One of these three compounds is also highly tolerated by human host cells. Findings from comparative susceptibility analyses in the CF0001-resistant variant and the wildtype strain predict ultralow rates of spontaneous mutation leading to resistance to this new specific antichlamydial in *C*. *trachomatis*.

## Results

### Fragments of CF0002 lack antichlamydial activities

**Fig 1:**
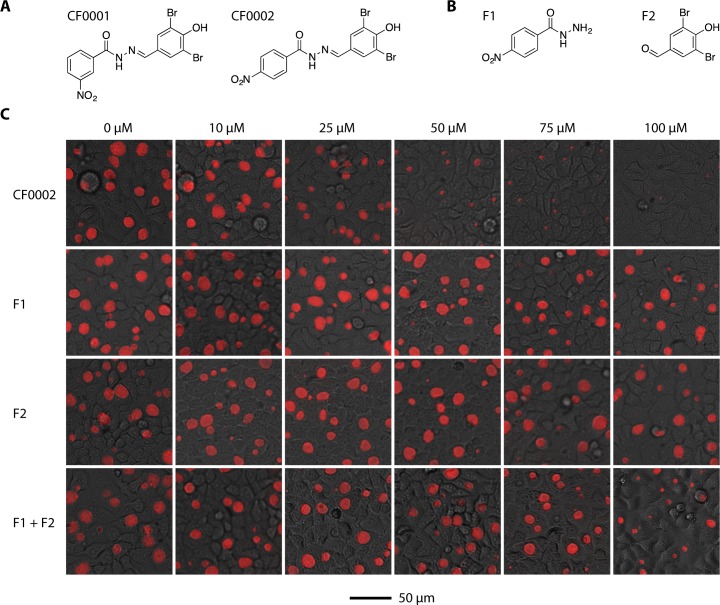
Lack of significant antichlamydial activities in fragments of CF0002. Structures of CF0001 and CF0002 (A) and hypothetic hydrolytic products F1 and F2 of CF0002 (B). (C) Strong inhibition of *C*. *trachomatis* L2 growth by CF0002 but not F1 and/or F2. HeLa cells were infected with RFP/iGFP-L2r at a multiplicity of infection (MOI) of 0.2 inclusion-forming unit per cell. Chemical treatment started 1 h postinoculation. The 0 μM cultures contained 1% DMSO in their media. 28 h postinoculation, images of chlamydial inclusions emitting red fluorescence signals as well as cellular images under bright light were acquired. A scale bar is at the bottom.

Two N-acylhydrazones, CF0001 and CF0002 (Fig 1A), have been shown to act as specific *Chlamydia* inhibitors [20]. The mechanism underlying the inhibition is unknown. Since some, but not all, N-acylhydrazones act as prodrugs through hydrolysis into two fragments [21], we investigated the possibility that CF0002 inhibits *Chlamydia* through a hydrolytic product, F1 or F2 (Fig 1B). As expected, CF0002 demonstrated dose-dependent inhibitory effects on the number and/or the size of the *C*. *trachomatis* inclusion in HeLa cells starting at 25 μM; inclusions formed in the presence of 100 μM were barely detectable (Fig 1C top panel). However, chlamydial growth was only marginally affected by either F1 or F2, even at 100 μM (Fig 1C, row 2 and 3, respectively). When both F1 and F2 were added to cultures, a noticeably additive effect was only observed at 100 μM each (Fig 1C, row 4). Nonetheless, the inhibition by 100 μM F1 and 100 μM F2 combined was still weaker than that of 50 μM CF0002. These data do not support the notion that a hydrolytic product of either CF0001 or CF0002 is responsible for their antichlamydial activity.

### SF1, SF2 and SF3 are strong antichlamydials

**Fig 2:**
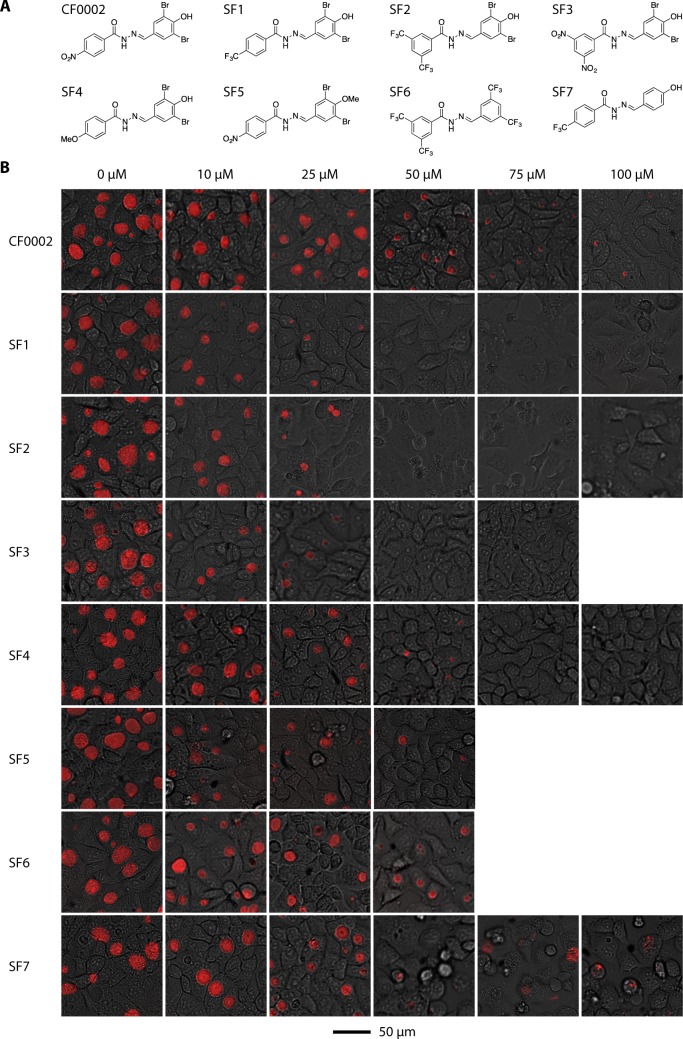
CF0002 analogs with strong antichlamydial activities. (A) Structures of CF0002 and new analogs. (B) Inclusions formed in cells treated with indicated N-acylhydrazone at specified concentrations. Experimental conditions and data collection were the same as in Fig 1C. Note the highest concentration tested for SF3, SF5 and SF6 was 75 μM, 50 μM and 50 μM, respectively, due to their limited solubility. The 0 μM cultures contained 1% DMSO in their media. A scale bar is at the bottom.

Since neither fragment of CF0002 showed significant antichlamydial activity, we explored modifications of CF0002 and determined the effects of seven analogs (Fig 2A) on *C*. *trachomatis* growth (Fig 2B). Four of the derivatives (SF1, SF2, SF4 and SF7) as well as CF0002 were tested at 0, 10, 25, 50, 75 and 100 μM whereas the highest concentration tested for SF3 was 75 μM, and those for SF5 and SF6 were 50 μM due to their limited solubility in the initial solvent DMSO and subsequently in the culture medium. The apparent minimal inhibition concentrations (MIC) of SF1, SF2 and SF3 were 50 μM. In contrast, 100 μM CF0002 failed to fully prevent inclusion formation (Fig 2B). Compared with SF1, SF2 and SF3, SF4 demonstrated a slightly lower antichlamydial activity with an MIC of 75 μM. SF5 appeared to be as effective as SF4, whereas SF6 appears to be as effective as CF0002. However, the MIC for neither SF5 nor SF6 could be determined because their highest concentration tested could not exceed 50 μM. Finally, SF7 appears to be as effective as or less than CF0002 (Fig 2B).

**Fig 3:**
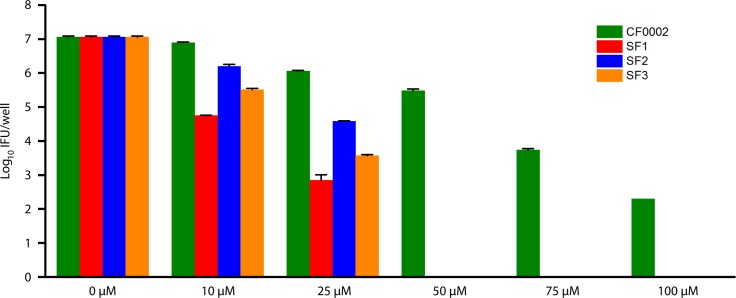
Stronger chlamydicidal activities in SF1, SF2 and SF3, as compared to CF0002. HeLa cells were infected with RFP/iGFP-L2r at an MOI of 0.2 inclusion-forming unit per cell. Chemical treatment started 1 h postinoculation. 40 h postinoculation, culture media were removed; cell lysates were prepared, subjected to 1:10 serial dilution and inoculated onto L929 monolayers. Infected L929 cells were cultured in the presence of anhydrotetracycline to induce GFP expression. GFP-expressing inclusions were enumerated 30 h later. Data are averages ± standard deviations of duplicate experiments. Single and double asterisk denote statistically significant differences between N-acylhydrazone-treated cultures and control DMSO-treated cultures (P < 0.05 and P < 0.01, respectively, two-tailed Student’s t test).

We further quantified recoverable inclusion forming units (IFUs) from the infected cells treated with CF0002, SF1, SF2, SF3 or DMSO. These experiments revealed the minimal chlamydicidal concentrations (MCC) for SF1, SF2 and SF3 were 50 μM, whereas the MCC of CF0002 was higher than 100 μM (Fig 3). Taken together, data in both Fig 2 and Fig 3 demonstrate that SF1, SF2 and SF3 are more potent antichlamydials than CF0002.

### CF0001- and CF0002-resistant *C*. *muridarum* variant MCR is cross-resistant to SF1, SF2 and SF3

**Fig 4:**
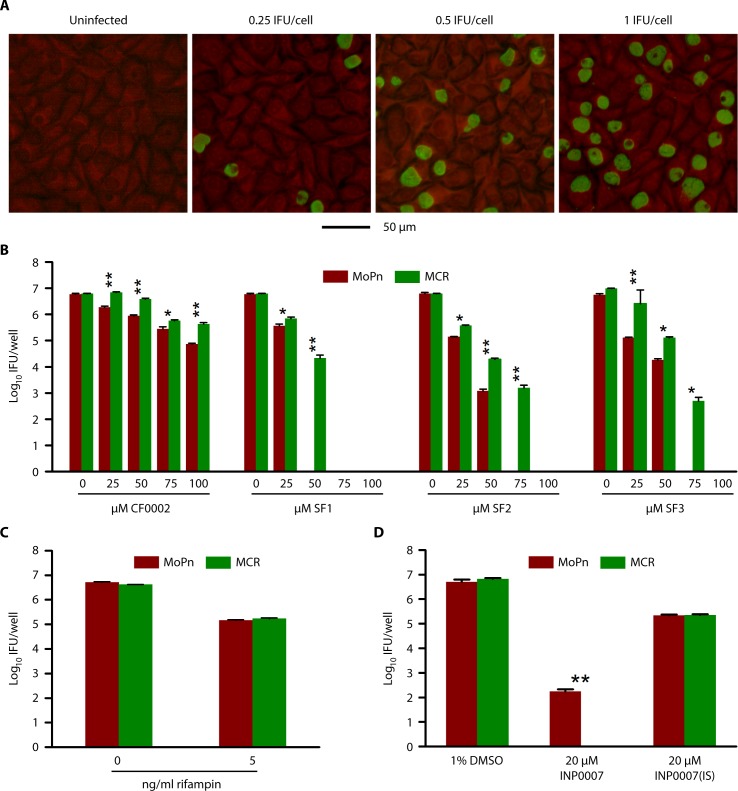
Differential susceptibilities to SF1, SF2 and SF3 in wildtype *C*. *muridarum* strain MoPn and the CF0001- and CF0002-resistant variant MCR. (A) L929 cells were either uninfected or infected with increased doses of MoPn EBs. 22 h postinoculation, cells were fixed, and subjected to immunofluorescence staining using a polyclonal mouse anti-MoPn antibody. A scale bar is at the bottom. (B-D) HeLa cells were infected with MoPn or MCR at a multiplicity of infection (MOI) of 0.2 inclusion-forming unit per cell. Chemical treatment started 1 h postinoculation. 24 h postinoculation, culture media were removed. Cell lysates were prepared, subjected to 1:10 serial dilution, and inoculated onto L292 cells. 22–24 h later, infected cells were fixed and chlamydial inclusions were stained with an immunofluorescence assay using the same antibody as in (A). (B) Compared to wildtype *C*. *muridarum* MoPn, MCR showed increased tolerance to CF0001, SF1, SF2 and SF5. (C) MoPn and MCR showed comparable susceptibilities to rifampin. (D) MCR was more susceptible to INP0007 but not iron-saturated INP0007 [INP0007(IS)]. (B-D) The 0 μM cultures contained 1% DMSO in their media. Data are averages ± standard deviations of duplicate experiments. Single and double asterisk denote statistically significant differences between MoPn and MCR (P < 0.05 and P < 0.01, respectively, two-tailed Student’s t test).

We next compared inhibition efficiencies of SF1, SF2 and SF3 in wildtype *C*. *muridarum* MoPn with their inhibition efficiencies in MCR to infer their antichlamydial mechanisms. MCR is an isogenic variant of MoPn, which is partially resistant to CF0001 and CF0002 [20]. The inhibition was determined by quantifying infectious EBs through immunofluorescence staining using a polyclonal anti-MoPn antibody (Fig 4A). Consistent with data obtained with *C*. *trachomatis* (Figs 2 & 3), SF1, SF2 and SF3 all demonstrated stronger antichlamydial activities than CF0002 for MoPn (Fig 4B). Noticeably, the new inhibitors consistently inhibited MCR less efficiently, as compared with MoPn. Nonetheless, they still fully abrogated MCR’s capacity to form progeny EBs at 75 μM (SF1 and SF3) or 100 μM (SF2) (Fig 4B).

To determine whether MCR is generally resistant to antibacterials, we determined the inhibition efficiencies of rifampin and INP0007 in MCR and MoPn. Whereas rifampin inhibits bacterial RNA synthesis [22], INP0007 interferes with chlamydial heme metabolism and also affects iron metabolism in the host cell [23]. While MoPn and MCR were equally susceptible to rifampin (Fig 4C), MCR was significantly more susceptible to INP0007 (Fig 4D), despite its lower susceptibility to CF0001, SF1, SF2 and SF3 (Fig 4A). Interestingly, MoPn and MCR were equally susceptible to iron-saturated INP0007 (Fig 4D), which presumably only interferes with chlamydial heme metabolism without affecting iron availability in the host cell. Consistent with published studies of INP compounds [23, 24], iron-saturated INP0007 at 20 μM was about 100 fold a weaker MoPn inhibitor than INP0007 (Fig 4D). Taken together, the data in Fig 4 indicate that SF1, SF2 and SF3 likely share the same antichlamydial mechanism as CF0002 (and CF0001).

### SF3 is highly tolerated by mammalian cells

**Fig 5:**
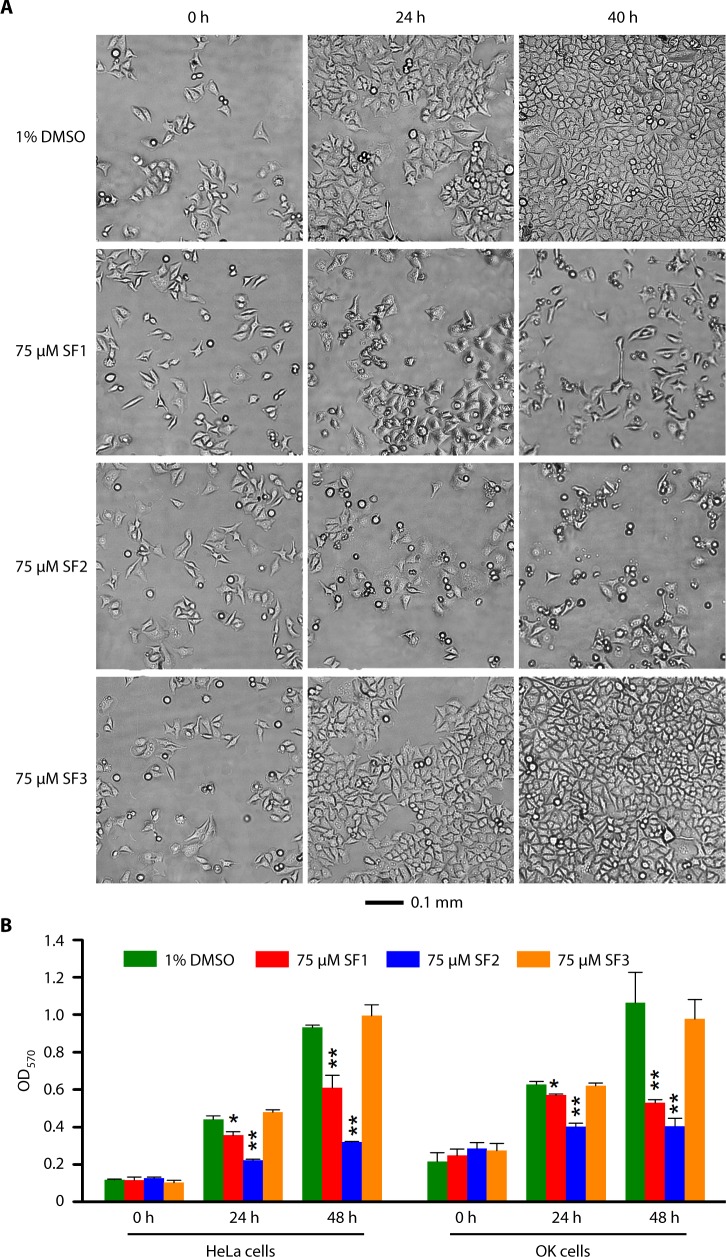
Lack of toxic effects of SF3 on mammalian cells. (A) SF1 and SF2 but not SF3 apparently inhibited cell growth and cause morphological changes. HeLa cells at low confluency were treated with SF1, SF2, SF3 or the solvent DMSO. Images were acquired at indicated times following chemical treatment. A scale bar is at the bottom. (B) Adverse effects of SF1 and SF2 but not SF3 on viability of HeLa and OK cells were detected using the MTT assay.

To determine effects of SF1, SF2 or SF3 on host cells, we cultured HeLa (human cervical carcinoma) cells and OK (immortalized but nonmalignant opossum kidney tubule epithelial cells) with media containing the new antichlamydials starting with low cell confluency. Under microscope, HeLa cells and OK cells demonstrated similar responses. Images of HeLa cells at 0, 24 and 40 h treatment are presented in Fig 5A. Compared to 1% DMSO, both 75 μM SF1 and 75 μM SF2 halted cell growth. SF2 also caused a significant proportion of cells to round up. In contrast, SF3-treated cells looked indistinguishable from control DMSO-treated cells. Results of MTT assay, which quantitatively measures metabolic activity of cells and is predictive of cell viability, corroborated microscopic observations (Fig 5B). Thus, SF3 but not SF1 and SF2 lacks toxicity to mammalian cells.

### SF1, SF2 and SF3 do not inhibit *Lactobacillus* growth

Lactobacilli help protect the female genital tract from pathogens [25]. We determined the impact of SF1, SF2 and SF3 on the growth of two *L*. *crispatus* strains and one *L*. *jesenii* strain isolated from the vagina. Growth kinetics of all the three strains cultured in medium containing 100 μM SF1, 100 μM SF2 or 75 μM SF3 was indistinguishable from the growth kinetics of bacteria cultured in control medium (Fig 5). Similar results were also obtained with 500 μM SF1 and 500 μM SF2. As for SF3, no concentration higher than 75 μM was tested because of its limited solubility. These results suggest that like CF0001 and CF0002 [20], SF1, SF2 and SF3 inhibit chlamydiae without affecting the growth of vaginal probiotic lactobacilli even at very high concentrations.

### Isolation of resistant mutants after selection with rifampin and spectinomycin but not SF3

We assessed rates of random mutations leading to resistance to SF3, rifampin and spectinomycin. Selection of rifampin- and spectinomycin-resistant mutants was initiated with a T75 flask of HeLa cells and 10^7^ IFU of MoPn EBs for each inhibitor. The MIC of rifampin was 8 ng/ml. Selection for rifampin-resistant variants was performed with 6 ng/ml of the antibiotic. Inclusions ceased to be visible in the culture of the 7^th^ passage, but a few inclusions reappeared in the culture of the 8^th^ passage. Chlamydiae from this last passage were found to tolerate 32 ng/ml rifampin. Sequencing analyses revealed two nucleotide substitutions within a single codon (CAG → TAC) in the RNA polymerase β subunit gene (*rpo*B) in all 4 clonal populations. This specific codon switch translates to Q455Y substitution in the RpoB protein. The RpoB protein is an established target of rifampin, and mutations in this region of RpoB have been shown to cause rifampin-resistance in other bacteria [26].

The MIC of spectinomycin was 50 μg/ml. Spectinomycin-resistant variants were selected for at an inhibitor concentration 10 μg/ml. Inclusions became progressively fewer from passage 1 through passage 6, and completely undetectable in the culture of the 7^th^ passage. After another passage with 10 ug/ml spectinomycin, 2 additional blind passages with inhibitor-free medium, inclusions reemerged. The remerging chlamydiae were found to tolerate at least 100 μg/ml spectinomycin. Sequencing of the 16S ribosomal RNA gene, a known target of spectinomycin, reveal two independent variants. One had a G→T transversion at nucleotide 134907, while the other had a C→T transition at nucleoside 135035 (as numbered in the reference genome, GenBank accession number CP007276).

Our previous studies demonstrated low rates of resistance to CF0001 and CF0002 [20]. Since the structure of SF3 (Fig 2A) closely resembles the structures of CF0001 and CF0002 (Fig 1A), we predicted that frequency of random mutation rendering resistance to SF3 is also low. Therefore, we initiated selection of SF3-resistant variants with two T150 flasks and 4 X 10^7^ IFU of MoPn EBs (i.e., 4 times of the number of initial EBs that were used for the selection of rifampin- and spectinomycin-resistant variants). After successive passages with SF3 at 10 μM and 30 μM SF3 for 7 and 4 passages, respectively, no inclusions formed when the concentration increased to 50 μM, the MIC (Fig 4). No inclusions reemerged after four successive blind passages in medium free of the inhibitor. Taken together, the results of the resistant variant selection experiments support the notion that rates of resistance to SF3 is low in MoPn.

## Discussion

**Fig 6:**
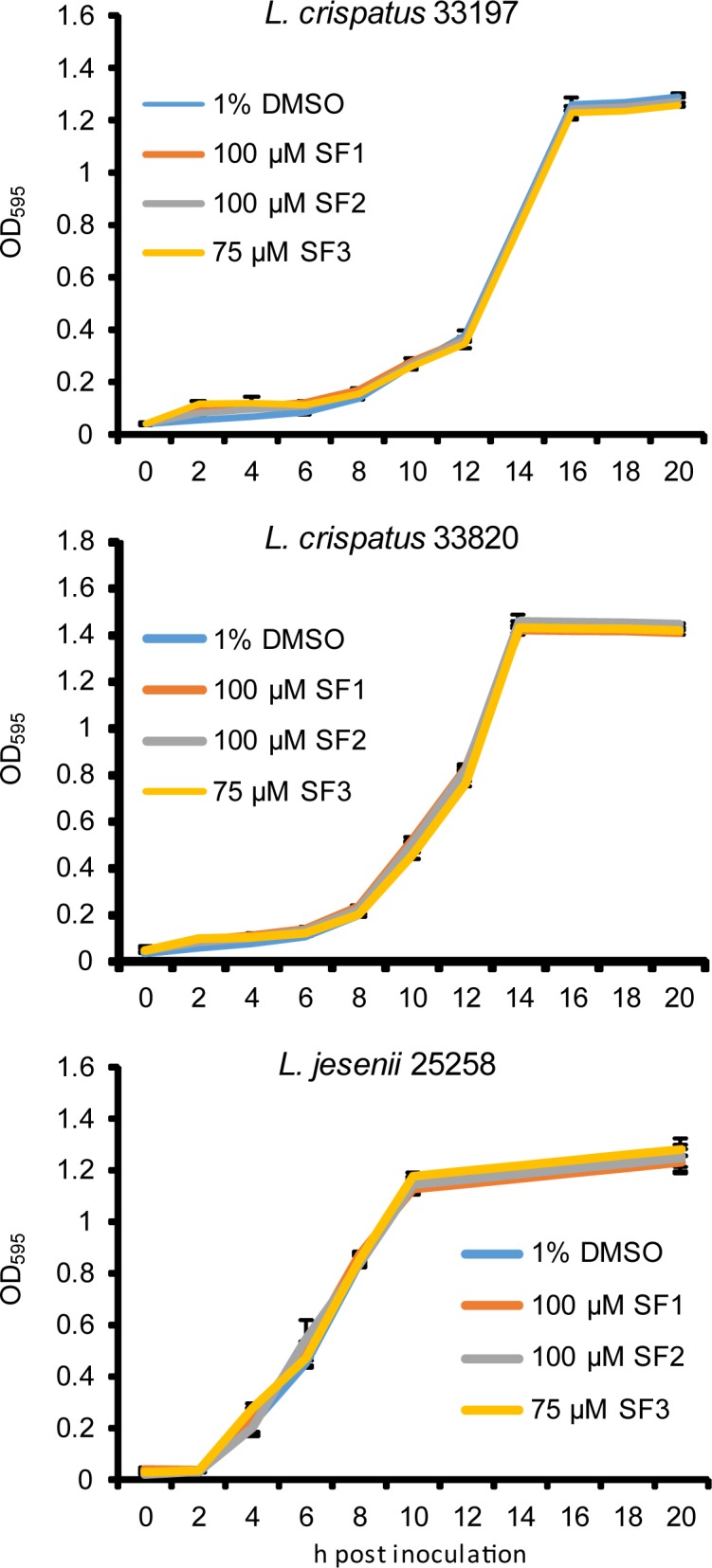
No effects of SF1, SF2 and SF3 on growth of vaginal *Lactobacillus* spp. Overnight cultures were diluted 1:100 with fresh MRS broth containing 100 μM SF1, 100 μM SF2, 75 μM SF3 or 1% DMSO. OD_595_ values were recorded at indicated h postinoculation. Values are averages ± standard deviations of triplicate experiments.

Whereas ocular-tropic *C*. *trachomatis* remains the number one infectious cause of blindness in developing countries, urogenital-tropic *C*. *trachomatis* is unquestionably the most common sexually transmitted bacterial pathogen worldwide, and arguably the most common of all sexually transmitted pathogens. In this study, we have identified three new N-acylhydrazones (SF1, SF2 and SF3) with strong antichlamydial activities (Fig 2 and Fig 3). All three compounds are well tolerated by beneficial vaginal lactobacilli (Fig 6). SF3 is also well tolerated by host human cells (Fig 5). Thus, SF3 appears to be a safe and specific antichlamydial.

There are at least three rationales for developing new antichlamydials. First, although antibiotic resistance is currently not a clinical problem, it may become one in the future. Indeed, in the US and Europe, tetracycline-resistance is already widespread in *Chlamydia seus*, a porcine pathogen, following decades’ long use of tetracycline by farmers to prevent bacterial infection and promote growth of pigs [27–29]. Isolation of two *C*. *trachomatis* strains resistant to multiple antibiotics (doxycycline, azithromycin and ofloxacin) from three patients (two of whom were husband and wife) further supports the risk of emergence of antibiotic-resistant human chlamydiae [30]. Fortunately, these multiple-resistant strains did not seem to spread further.

The second rationale for developing new antichlamydials is that treating chlamydial infection with current therapeutics may facilitate the occurrence of antibiotic resistance in other pathogens. Although only a small portion of people infected with *C*. *trachomatis* (and *C*. *pneumoniae*) are diagnosed and treated with antibiotics, the absolute number of infected people who receive antibiotics is still of significance, which can be regarded as an effective driver for antibiotic resistance in other pathogens. For this reason, *Chlamydia-*specific antibiotics are preferred, and likely will extend the “use lives” of existing antibiotics for some pathogens even though several lethal pathogens are already completely resistant to almost all clinical drugs.

The third rationale for developing specific antichlamydials relates to the adverse effects of broad-spectrum antibiotics on microbiotas. In most reproductive-age women, lactobacilli dominate their vaginal microbiota. Abundant evidence suggests that vaginal lactobacilli such as *L*. *crispatus* are required for the health of the genital tract in women. By producing lactic acid, vaginal lactobacilli maintain an acidic vaginal environment with a pH range of 2.8–4.2. Loss of vaginal lactobacilli, for example, due to broad spectrum antibiotics, may cause vaginal dysbiosis, leading to yeast vaginosis [31–36]. SF1, SF2 and SF3 are all well tolerated by vaginal lactobacilli (Fig 6). However, how they would affect other microbiotas, particularly the gut microbiota, as well as other beneficial components of the vaginal microbiota is not known. Addressing this question with certainty will require human studies for two reasons. First, most microbiota components are not cultivable. Second, humans and animals differ in microbiota composition substantially. For example, most animals have a near neutral vaginal pH. There are lactic-acid producing microbes in the lower genital tract of some non-human primates, but their vaginal pH is significantly higher than that of women.

The rate of spontaneous mutation leading to resistance is a critical aspect for antimicrobials. Previously, although a lengthy three month selection of MoPn resulted in the isolation of a partially CF0001/CF0002-resistant variant MCR, repeated efforts to isolate additional resistant variants even from *C*. *trachomatis* and *C*. *muridarum* stocks that were pretreated with the mutagen ethyl methanesulfonate failed. Compared with MoPn, MCR has four single nucleotide polymorphisms in the genome, which affect four different genes, thus suggesting that multiple mutations may be required for resistance to these inhibitors, and that MCR is an extremely rare variant [20]. SF3 (Fig 2A) has a high degree of structural resemblance with CF0001 and CF0002 (Fig 1A), suggesting that SF3 shares the same inhibition mechanism as CF0001 and CF0002 (even though the exact mechanism has yet to be defined), and therefore it would also be difficult for *Chlamydia* to develop resistance to SF3. Two additional lines of evidence support this proposition. First, the CF0001/CF0002-resistant MCR is cross resistant to SF3 but not other types of antichlamydials (Fig 4). Second, whereas rifampin- and spectinomycin-resistant variants were obtained when 10^7^ IFU of EBs was used for each selection, no SF3-resistant variant was obtained from 4 X 10^7^ IFU.

It was previously shown that MCR has a growth defect at an early developmental stage [20]. It is also interesting that MCR is more susceptible to INP0007 (Fig 4D). Although INP0007 is an inhibitor of type III secretion of Gram-negative bacteria [37] and blocks chlamydial growth [38, 39], it has a moderate toxicity to host cells [20]. The host cell toxicity of INP0007 is related to its iron-chelating activity [23, 24]. Iron depletion is an innate immune response that is activated by microbial infections [40]. Since MCR and parental MoPn are equally susceptible to the antibacterial iron-saturated INP0007 (Fig 4D) and rifampin (Fig 4C), we hypothesize that, compared to MoPn, MCR is more susceptible to INP0007-medidated iron starvation in the host cells. Thus, it is likely that increased tolerance to specific antichlamydial N-acylhydrazones is linked to decreased fitness, particularly under iron starvation.

In conclusion, we have identified a new N-acylhydrazone (SF3) that acts as specific antichlamydial, is well-tolerated by host human cells, and is not harmful to beneficial vaginal lactobacilli. Importantly, SF3 appears to share the same inhibition mechanisms as CF0001 and CF0002, yet is significantly more potent and able to overcome chlamydial resistance to CF0001 and CF0002. Thus, it should be even more difficult for chlamydiae to develop resistance to SF3. Our findings also suggest that resistance to specific antichlamydial N-acylhydrazones is linked to decreased fitness, particularly when host cells are undergoing iron starvation, which is trigged often during microbial infection. Therefore, resistance to these inhibitors may be linked to decreased pathogenicity.

## Materials and methods

### Chemicals

(*E*)-*N*'-(3,5-dibromo-4-hydroxybenzylidene)-4-nitrobenzohydrazide (CF0002) and (*E*)-*N*'-(3,5,-dibromo-2-hydroxybenzylidene)-4-nitrobenzohydrazide (INP0007) were previously described. Iron saturated INP0007 [INP0007(IS)] was prepared by combining INP0007 and FeCl_3_ at an equal molar ratio one hour before use. 3,5-dibromo-4-hydroxybenzaldehyde (F2) and 3-(4,5-dimethylthiazol-2-yl)-2,5-diphenyltetrazolium bromide (MTT) were purchased from Sigma-Aldrich. Synthetic procedures and characterization data for 4-nitrobenzohydrazide (F1), (*E*)-*N*'-(3,5-dibromo-4-hydroxybenzylidene)-4-(trifluoromethyl)benzohydrazide (SF1), (*E*)-*N*'-(3,5-dibromo-4-hydroxybenzylidene)-3,5-bis(trifluoromethyl)benzohydrazide (SF2), (*E*)-*N*'-(3,5-dibromo-4-hydroxybenzylidene)-3,5-dinitrobenzohydrazide (SF3), (*E*)-*N*'-(3,5-dibromo-4-hydroxybenzylidene)-4-methoxybenzohydrazide (SF4), (*E*)-*N*'-(3,5-dibromo-4-methoxybenzylidene)-4-nitrobenzohydrazide (SF5), (*E*)-*N*'-(3,5-bis(trifluoromethyl)benzylidene)-3,5-bis(trifluoromethyl)benzohydrazide (SF6) and (*E*)-*N*'-(4-hydroxybenzylidene)-4-(trifluoromethyl)benzohydrazide (SF7) are provided as supporting information.

### Host cells and culture conditions

Human cervical carcinoma HeLa cells were used for chemical inhibition tests. Mouse fibroblast L929 cells were used as reporter cells for quantifying recoverable inclusion-forming units (IFU) of elementary bodies (EBs, the infectious chlamydial cells) from the inhibition tests as well as for raising *Chlamydia* EB stocks. Opossum kidney (OK) cells, in addition to HeLa cells, were used for toxicity experiments. All cell lines were maintained as adherent cultures using Dulbecco-modified Eagle’s medium containing 5% (L929 and OK) or 10% (HeLa) fetal bovine serum and 20 μg/ml gentamicin. They were cultured in 37°C incubators with humidified air supplemented with 5% CO_2_.

### *Chlamydia* strains

RFP/iGFP-L2r was derived by transforming a plasmid-free *C*. *trachomatis* variant, named L2R [l2(25667R)] [41], with the shuttle vector pASK-GFP/mKate2-L2 [42] as previously described [43]. The transformation resulted in restoration of the *C*. *trachomatis* plasmid-encoded genes, constitutive expression of mKate, a red fluorescence protein (RFP) and expression of a green fluorescence protein that is induced with anhydrotetracycline (iGFP). Wildtype *C*. *muridarum* (strain Nigg II, traditionally known as mouse pneumonitis pathogen or MoPn) was originally purchased from ATCC [44]. MCR, an MoPn variant with a low level of resistance to CF0001 and CF0002, was previously described [20]. EB stocks were raised from L929 cells and purified with ultracentrifugation through MD-76 gradients [45].

### *Chlamydia* inhibition tests

Potential antichlamydial activities in small compounds were evaluated by determining their effects on formation of chlamydial inclusions and/or progeny EBs as previously reported [20, 46, 47]. At the time of inoculation, HeLa cells were about 70% confluent. The multiplicity of infection was 0.2 inclusion-forming unit (IFU) per cell. Chemical treatment was initiated by replacement of the culture medium with fresh medium containing indicated concentrations of an inhibitor or the vehicle DMSO (final concentration: 1%) 1 h postinoculation. To determine effects of compounds on inclusion formation, life cultures of RFP/iGFP-L2r-infected life were imaged 28 h postinoculation using an Olympus monochrome CCD camera under an Olympus IX51 fluorescence microscope through the red fluorescence channel. Corresponding bright-light images were also obtained. Image processing (coloring and imaging overlay) were accomplished by using the PictureFrame software [43]. Lowest concentration of a chemical that resulted in apparent absence of chlamydial inclusion formation was defined as the minimal inhibition concentration (MIC). To determine effects of compounds on progeny RFP/iGFP-L2r EB formation, media were aspirated. Cells were scraped off the plastic, collected into 200 μL sucrose-phosphate-glutamic acid buffer and disrupted by sonication 40 h postinoculation [47]. Cell lysates were clarified by centrifugation (500 g, 10 min). Resulting supernatants were subjected to 1:10 serial dilution, and inoculated to L929 monolayers at about 90% confluency in 96-well plates. Infected L929 cells were cultured in medium containing 1 μg/mL cycloheximide to maximize chlamydial growth. 20 h postinoculation, anhydrotetracycline was added to culture medium (final concentration: 20 nM) to induce GFP expression. Green fluorescence inclusions were enumerated 30 h postinoculation under an Olympus XI-51 fluorescence microscope following fixation sequentially with paraformaldehyde and methanol as detailed previously [48]. To determine effects of compounds on progeny EB formation for MoPn- and MCR, infected cells were collected and lysed 22–24 h postinoculation. Lysates were inoculated onto L929 cells. Following 24 h incubation in medium containing 1 μg/mL cycloheximide, cells were fixed with cold methanol, and reacted sequentially with pooled sera collected from mice infected with MoPn (Kang and Fan, unpublished studies) at 1:4,000 dilution and fluorescein isothiocyanate-conjugated rabbit anti-mouse IgG (Sigma-Aldrich). Inclusions were numerated as described above. For both *C*. *trachomatis* and *C*. *muridarum*, the lowest concentration of a compound that resulted in full abrogation of progeny EB formation was defined as the minimal chlamydicidal concentration (MCC).

### Determination of host cell toxicity

Host cell toxicity of antichlamydials was assessed by visualizing cell growth with light microscopy and by measuring cellular metabolic activities using an MTT [3-(4,5-dimethylthiazol-2-yl)-2,5-diphenyltetrazolium bromide] assay [49]. To visualize effects of compounds on cell growth, HeLa cells were seeded onto 12-well plates at 25% confluency. Treatment with antichlamydials or DMSO was initiated 5 h after seeding. At indicated times following addition of chemical compounds, cell densities were observed under a light microscope, and phase contrast images were acquired.

MTT assays were carried out using 96-well plates. HeLa and OK cells were seeded at 10% and 20% confluency, respectively. After 3 h incubation at a tissue culture incubator, the culture medium was replaced with 90 μL (per well) phenol-red-free DMEM containing 10% fetal bovine serum and 75 μM SF1, SF2 or SF3, or 1% DMSO. At indicated times, 10 μL of a 12 mM MTT stock solution prepared in phosphate-buffered saline was added into each well. Cells were cultured for additional 4 h, and lysed by addition of 100 μL 10% (W/V) sodium dodecyl sulfate containing 10 mM HCl. After another 4 h incubation at 37°C, plates were placed on an orbital shaker for 5 min and OD_570_ values were obtained using a plate reader.

### Determination of tolerance by *Lactobacillus*

*L*. *crispatus* strains ATCC33197 (*L*. *crispatus* 33197) and ATCC33820 (*L*. *crispatus* strains ATCC33820) and *L*. *jesenii* strain ATCC25258 (*L*. *jesenii* 25258) were cultured with the MRS Lactobacilli broth (Sigma) in a humidified 5% CO_2_ incubator [47]. For testing the effects of antichlamydials on lactobacilli, overnight cultures were diluted 1:100 with fresh MRS broth containing an inhibitor or vehicle DMSO on 96-well plates. OD_595_ was measured on a plate reader at indicated times.

### Selection for resistant variants and isolation of clonal populations

Basic procedures for selection of with resistance to inhibitors have been previously described [20, 46, 47]. Clonal populations were generated from resistant chlamydiae by limiting dilution [50].

### DNA sequencing

Sequences of the RNA polymerase β subunit gene (*rpo*B) and the 16S ribosomal RNA gene as well as their flanking regions in clones resistant to rifampin and spectinomycin, respectively, were determined using the automated fluorochrome-conjugated dideoxynucleotide termination sequencing technique through paid service provided by Macrogen USA [20, 46].

## Supporting information

S1 File (PDF)
