# Supplementary material for: Identification of a strong and specific antichlamydial N-acylhydrazone
Source: PLoS One. 2017 Oct 3;12(10):e0185783. doi: 10.1371/journal.pone.0185783 (PMC5626472; doi:10.1371/journal.pone.0185783)
Supplement: S1 File — (PDF) [file pone.0185783.s001.pdf]

Identification of a Strong and Specific Antichlamydial N-Acylhydrazone

Huirong Zhang[1], Anuj Kunadia[2], Yingfu Lin[2], Joseph D. Fondell[1],
Daniel Seidel[2]*, and Huizhou Fan[1]*

[1] *Department of Pharmacology, Robert Wood Johnson Medical School, Rutgers, The State University of New Jersey, Piscataway, New Jersey, USA*
[2] *Department of Chemistry and Chemical Biology, School of Arts and Sciences, Rutgers, The State University of New Jersey, Piscataway, New Jersey, USA*

Supporting Information

I. General Information

Reagents and solvents were purchased from commercial sources and were used directly. Analytical thin layer chromatography was performed on EM Reagent 0.25 mm silica gel 60 F_{254} plates. Visualization was accomplished with UV light and iodine stain. Melting points were recorded on a Thomas Hoover capillary melting point apparatus and are uncorrected. Infrared spectra were recorded on an ATI Mattson Genesis Series FT-Infrared spectrophotometer. Proton nuclear magnetic resonance spectra (^{1}H-NMR) were recorded on Varian VNMRS-500 MHz, 400 MHz, and 300 MHz instruments and are reported in ppm using $(CD_3)_2SO$ as an internal standard at 2.50 ppm. Data are reported as app = apparent, s = singlet, d = doublet, t = triplet, q = quartet, m = multiplet, comp = complex; integration; coupling constant(s) in Hz. Proton-decoupled carbon nuclear magnetic resonance spectra (^{13}C-NMR) were recorded on Varian VNMRS-500 MHz, 400 MHz, and 300 MHz instruments and are reported in ppm using $(CD_3)_2SO$ as an internal standard at 39.52 ppm. Mass spectra were recorded on a Finnigan LCQ-DUO mass spectrometer.

II. Preparation and characterization of 4-nitrobenzohydrazide

4-Nitrobenzohydrazide (F1): Following a known procedure,[1] to a stirring solution of 4-nitrobenzoyl chloride (1.0 g, 5.4 mmol, 1 equiv) in methanol (2.67 mL, 2.0 M) at room temperature was added dropwise hydrazine monohydrate (0.56 g, 0.54 mL, 11.3 mmol, 2.1 equiv). The reaction mixture was heated under reflux for 30 min and was then allowed to cool to room temperature. The resulting precipitate was collected by filtration, washed with water, and recrystallized from ethanol to yield 4-nitrobenzohydrazide (0.46 g, 47%). The characterization data matched those reported in the literature.[1]

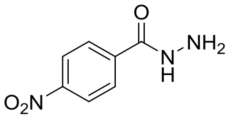

III. Preparation and characterization of *N*-acylhydrazone compounds

General procedure for the synthesis of *N*-acylhydrazones:

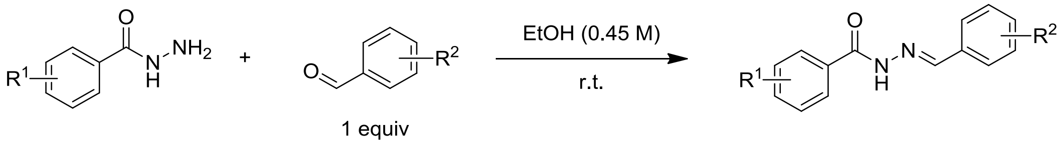

The synthesis of these compounds was adopted from a literature procedure.[2] To a solution or suspension of the corresponding benzohydrazide (1 equiv) in anhydrous ethanol (0.45 M) at room temperature was added the respective aldehyde (1 equiv). The resulting mixture was stirred at room temperature for the indicated amount of time. The resulting precipitate was collected by filtration and washed with cold water, ethanol, and ether, followed by drying under high vacuum to yield the product as a solid.

(*E*)-*N*'-(3,5-Dibromo-4-hydroxybenzylidene)-4-(trifluoromethyl)benzohydrazide (SF1): Following the general procedure, a mixture of 4-(trifluoromethyl)benzohydrazide[1] (1.68 mmol) and 3,5-dibromo-4-hydroxybenzaldehyde (1.68 mmol) was stirred for 1 hour. Compound **SF1** was isolated as a white solid in 53% yield (0.41 g); mp = 246–247 °C; R_f = 0.6 (CH_2Cl_2/MeOH 90:10 v/v); IR (film) 2923, 2853, 1459, 1377, 1308, 1161, 963, 722 cm^{-1}; ^{1}H-NMR (400 MHz, $(CD_3)_2SO$) δ 12.13 (s, 1H), 10.47 (s, 1H), 8.31 (s, 1H), 8.10 (d, J = 7.7 Hz, 2H), 7.97 – 7.82 (comp, 4H); ^{13}C-NMR (100 MHz, $(CD_3)_2SO$) δ 161.99, 152.32, 145.54, 137.08, 131.55 (q, J_{C-F} = 31.7 Hz), 130.75, 128.71, 128.59, 123.87 (q, J_{C-F} = 272.5 Hz), 125.44 (q, J_{C-F} = 3.8 Hz), 112.20; *m/z* (ESI-MS) 463.1 (^{79}Br, ^{79}Br) $[M – H]^-$, 465.0 (^{79}Br, ^{81}Br) $[M – H]^-$, 467.0 (^{81}Br, ^{81}Br) $[M – H]^-$.

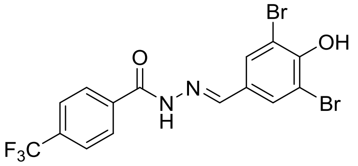

(*E*)-*N*'-(3,5-Dibromo-4-hydroxybenzylidene)-3,5-bis(trifluoromethyl)benzohydrazide (SF2): Following the general procedure, a mixture of 3,5-bis(trifluoromethyl)benzohydrazide[1] (0.99 mmol) and 3,5-dibromo-4-hydroxybenzaldehyde (0.99 mmol) was stirred for 16 hours. Compound **SF2** was isolated as a white solid in 51% yield (0.27 g); mp > 250 °C; R_f = 0.7 (CH_2Cl_2/MeOH 90:10 v/v); IR (film) 3404, 2923, 2853, 1638, 1460, 1377, 1279, 1157, 967, 722 cm^{-1}; ^{1}H-

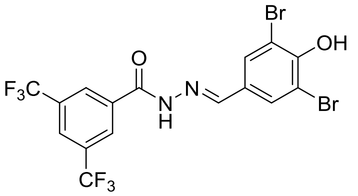

NMR (400 MHz, $(CD_3)_2SO$) δ 12.29 (s, 1H), 10.51 (br s, 1H), 8.54 (s, 2H), 8.38 (s, 1H), 8.31 (s, 1H), 7.95 (s, 2H); ^{13}C-NMR (100 MHz, $(CD_3)_2SO$) δ 160.21, 152.51, 146.11, 141.92, 135.54, 130.83, 130.53 (q, $J_{C\text{-}F}$ = 33.3 Hz), 128.43, 125.25, 123.07 (q, $J_{C\text{-}F}$ = 272.8 Hz), 112.17; *m/z* (ESI-MS) 531.1 (^{79}Br, ^{79}Br) $[M – H]^-$, 533.0 (^{79}Br, ^{81}Br) $[M – H]^-$, 535.0 (^{81}Br, ^{81}Br) $[M – H]^-$.

The structure of product **SF2** was confirmed by X-ray crystallography:

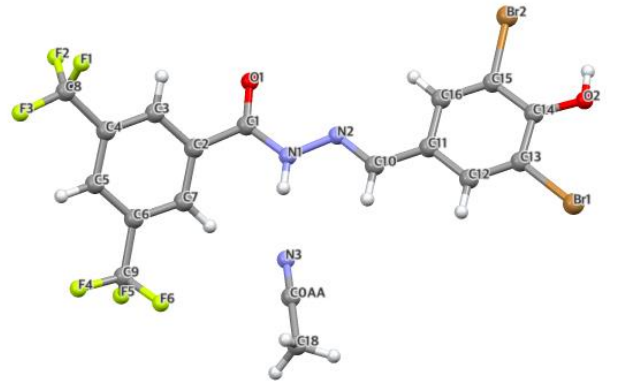

Compound **SF2** was crystallized from acetonitrile through slow evaporation at room temperature. The requisite CIF has been deposited with the CCDC (Deposition # 1548274).

(*E*)-*N*'-(3,5-Dibromo-4-hydroxybenzylidene)-3,5-dinitrobenzohydrazide (**SF3**): Following the general procedure, a mixture of 3,5-dinitrobenzohydrazide[3] (0.19 mmol) and 3,5-dibromo-4-hydroxybenzaldehyde (0.19 mmol) was stirred for 1.5 hours. Compound **SF3** was isolated as a yellow solid in 90% yield (82 mg); mp > 250 °C; R_f = 0.4 (CH_2Cl_2/MeOH 95:5 v/v); IR (film) 3424, 2923, 2853, 2127, 1644, 1541, 1460, 1377, 1347, 1168, 722 cm^{-1}; ^{1}H-NMR (500 MHz, $(CD_3)_2SO$) δ 12.49 (s, 1H), 10.54 (br s, 1H), 9.12 – 9.09 (m, 2H), 9.01 – 8.98 (m, 1H), 8.33 (s, 1H), 7.94 (s, 2H); ^{13}C-NMR (125 MHz, $(CD_3)_2SO$) δ 159.05, 152.55, 148.14, 146.56, 135.81, 130.92, 128.37, 127.92, 121.29, 112.19; *m/z* (ESI-MS) 485.1 (^{79}Br, ^{79}Br) $[M – H]^-$, 487.0 (^{79}Br, ^{81}Br) $[M – H]^-$, 488.9 (^{81}Br, ^{81}Br) $[M – H]^-$.

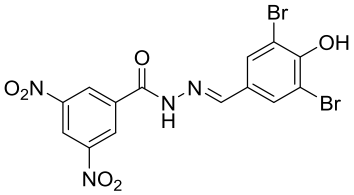

(*E*)-*N*'-(3,5-Dibromo-4-hydroxybenzylidene)-4-methoxybenzohydrazide (SF4): Following the general procedure, a mixture of 4-methoxybenzohydrazide[1] (1.03 mmol) and 3,5-dibromo-4-hydroxybenzaldehyde (1.03 mmol) was stirred for 16 hours. Compound **SF4** was isolated as a white solid in 88% yield (0.39 g); mp = 230–231 °C; R_f = 0.7 (CH_2Cl_2/MeOH 95:5 v/v); IR (film) 2923, 2854, 1620, 1562, 1460, 1399, 1377, 1303, 1264, 1069, 1027, 976, 892, 722 cm^{-1}; ^{1}H-NMR (300 MHz, $(CD_3)_2SO$) δ 11.81 (s, 1H), 10.40 (s, 1H), 8.28 (s, 1H), 7.94 – 7.86 (m, 4H), 7.05 (d, J = 8.9 Hz, 2H), 3.83 (s, 3H); ^{13}C-NMR (75 MHz, $(CD_3)_2SO$) δ 162.54, 162.03, 152.02, 144.03, 130.49, 129.57, 129.05, 125.28, 113.68, 112.21, 55.43; *m/z* (ESI-MS) 425.0 (^{79}Br, ^{79}Br) $[M – H]^-$, 427.0 (^{79}Br, ^{81}Br) $[M – H]^-$, 429.0 (^{81}Br, ^{81}Br) $[M – H]^-$.

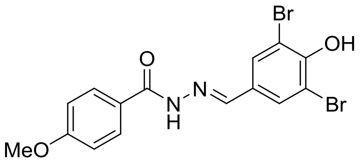

(*E*)-*N*'-(3,5-Dibromo-4-methoxybenzylidene)-4-nitrobenzohydrazide (SF5): Following the general procedure, a mixture of 4-nitrobenzohydrazide[1] (1.02 mmol) and 3,5-dibromo-4-methoxybenzaldehyde (1.02 mmol) was stirred for 72 hours. Compound **SF5** was isolated as an off-white solid in 29% yield (0.29 g); mp > 250 °C; R_f = 0.75 (CH_2Cl_2/MeOH 90:10 v/v); IR (film) 2923, 2854, 1664, 1522, 1460, 1377, 1270, 984, 721cm^{-1}; ^{1}H-NMR (500 MHz, $(CD_3)_2SO$) δ 12.35 (br s, 1H), 8.41 – 8.31 (comp, 3H), 8.15 (d, *J* = 8.2 Hz, 2H), 8.04 (s, 2H), 3.85 (s, 3H); ^{13}C-NMR (125 MHz, $(CD_3)_2SO$) δ 161.70, 154.62, 149.34, 145.15, 138.75, 133.30, 131.04, 129.26, 123.64, 118.11, 60.61; *m/z* (ESI-MS) 454.1 (^{79}Br, ^{79}Br) $[M – H]^-$, 456.0 (^{79}Br, ^{81}Br) $[M – H]^-$, 458.0 (^{81}Br, ^{81}Br) $[M – H]^-$.

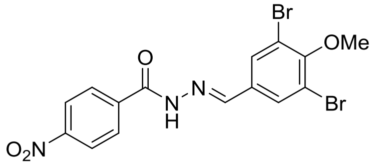

(*E*)-*N*'-(3,5-Bis(trifluoromethyl)benzylidene)-3,5-bis(trifluoromethyl)benzohydrazide (SF6): Following the general procedure, a mixture of 3,5-bis(trifluoromethyl)benzohydrazide[1] (2.94 mmol) and 3,5-bis(trifluoromethyl)benzaldehyde (2.94 mmol) was stirred for 1 hour. Compound **SF6** was isolated as a white solid in 66% yield (0.97 g); mp = 234.4–235 °C; R_f = 0.5 (Hexanes/EtOAc 70:30 v/v); IR (film) 2954, 2923, 2853, 1459, 1377, 1273, 1151, 723 cm^{-1}; ^{1}H-NMR (500 MHz, $(CD_3)_2SO$) δ 12.61 (s, 1H), 8.61 (s, 1H), 8.57 (s, 2H), 8.43 (s, 2H), 8.41 (s, 1H), 8.21 (s, 1H); Due to poor solubility of the compound, a ^{13}C-NMR was not obtained; *m/z* (ESI-MS) 495.1 $[M – H]^-$.

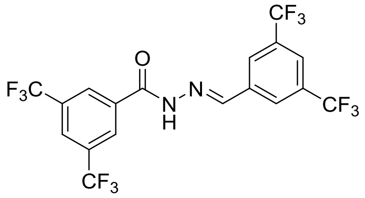

(*E*)-*N*'-(4-Hydroxybenzylidene)-4-(trifluoromethyl)benzohydrazide (**SF7**): Following the general procedure, a mixture of 4-(trifluoromethyl)benzohydrazide[1] (0.31 mmol) and 4-hydroxybenzaldehyde (0.31 mmol) was stirred for 72 hours. Compound **SF7** was isolated as a white solid in 15% yield (15 mg); mp > 250 °C; R_f = 0.25 (CH_2Cl_2/MeOH 95:5 v/v); IR (film) 3405, 2923, 2854, 1766, 1563, 1459, 1377, 1263, 859, 721 cm^{-1}; ^{1}H-NMR (300 MHz, $(CD_3)_2SO$) δ 11.83 (s, 1H), 9.96 (s, 1H), 8.36 (s, 1H), 8.10 (d, *J* = 8.1 Hz, 2H), 7.91 (d, *J* = 8.5 Hz, 2H), 7.58 (d, *J* = 8.5 Hz, 2H), 6.85 (d, *J* = 8.5 Hz, 2H); ^{13}C-NMR (75 MHz, $(CD_3)_2SO$) δ 161.64, 159.60, 148.91, 137.47, 131.36 (q, J_{C-F} = 32.2 Hz), 129.00, 128.46, 125.44 (q, J_{C-F} = 3.8 Hz), 125.09, 124.37 (q, J_{C-F} = 269.8 Hz), 115.74; *m/z* (ESI-MS) 307.2 $[M – H]^-$.

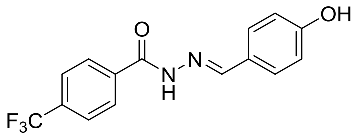

IV. NMR Spectra

^{1}H-NMR of **SF1** in $(CD_3)_2SO$:

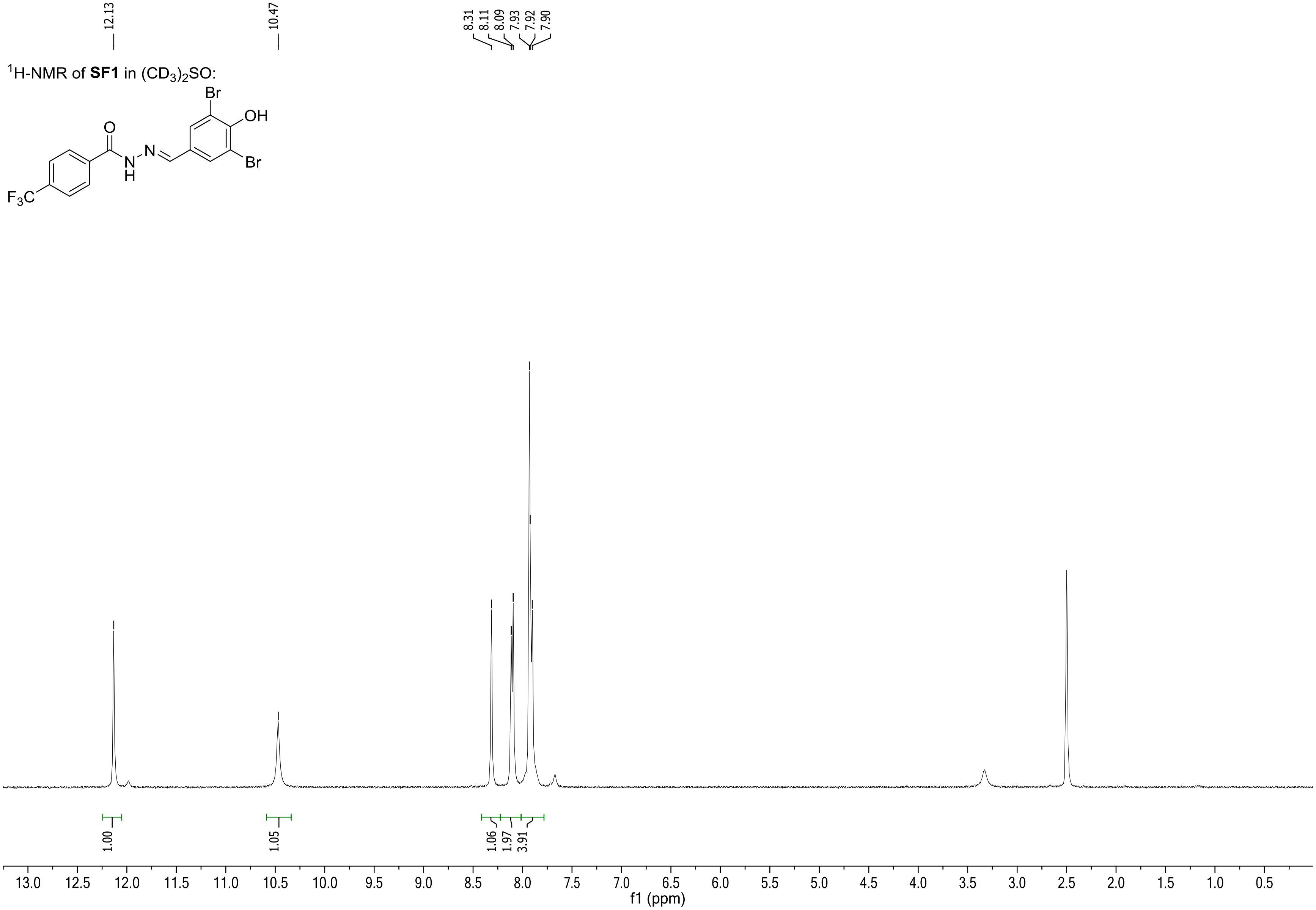

^{13}C-NMR of **SF1** in $(CD_3)_2SO$:

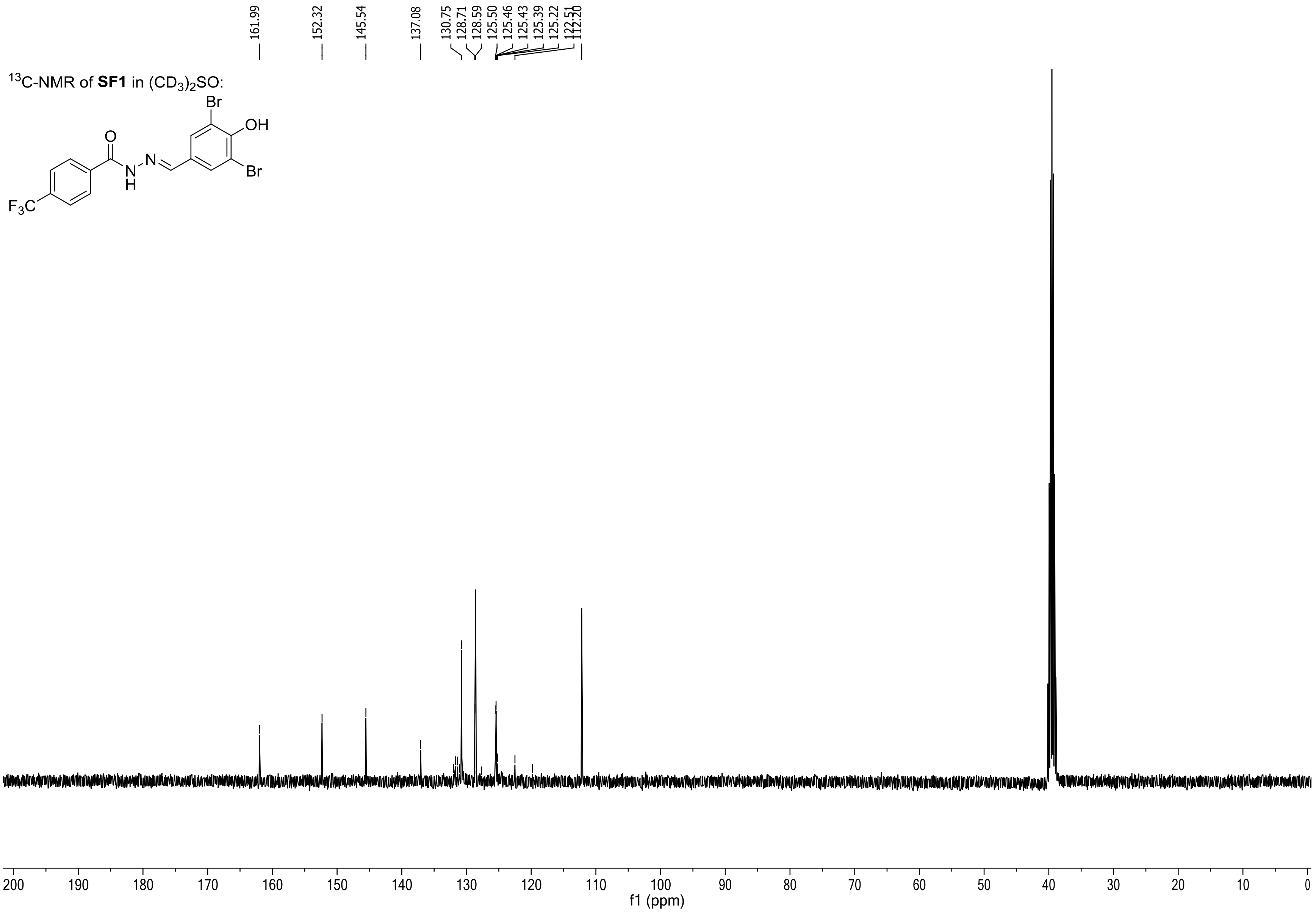

^{1}H-NMR of **SF2** in $(CD_3)_2SO$:

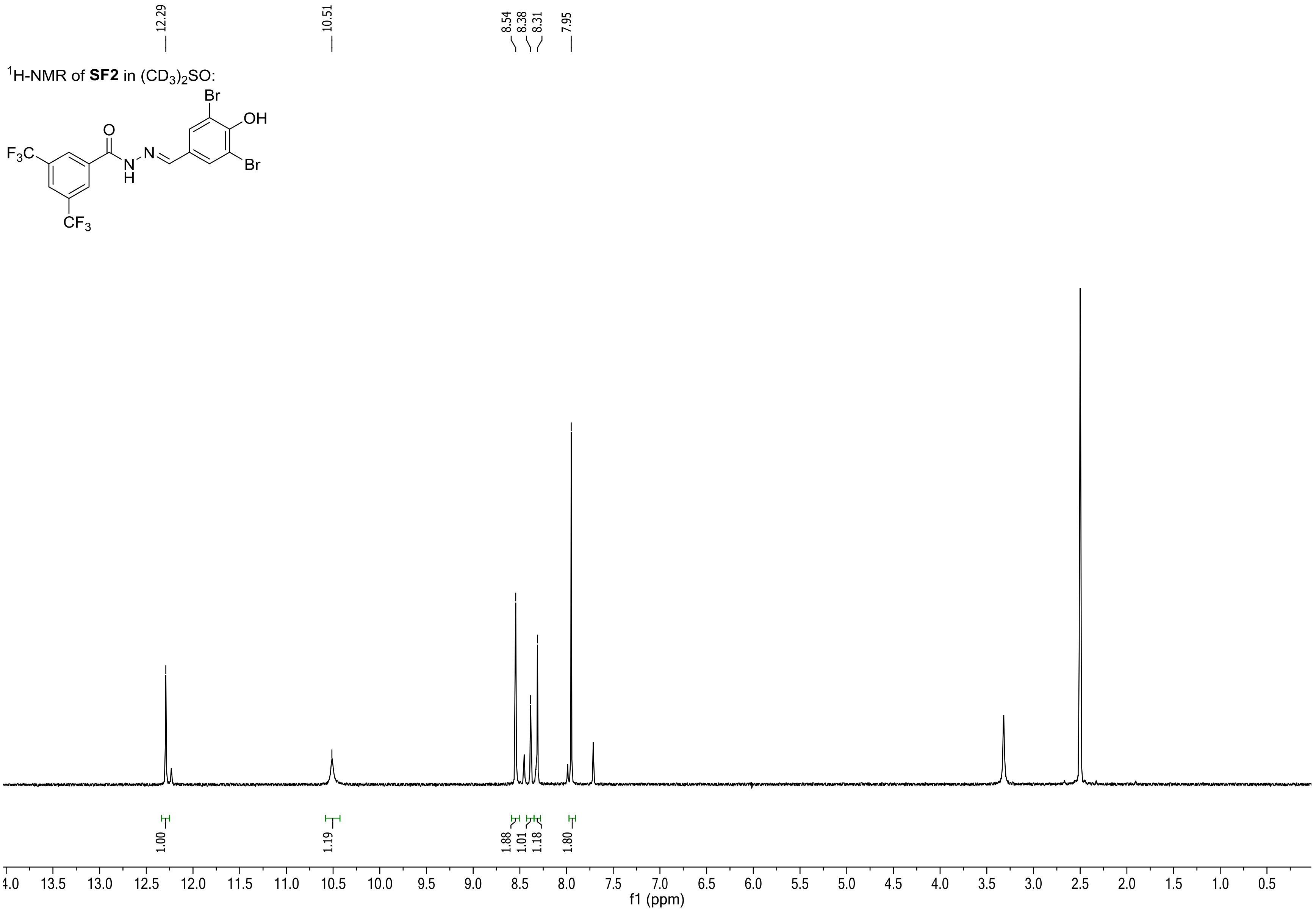

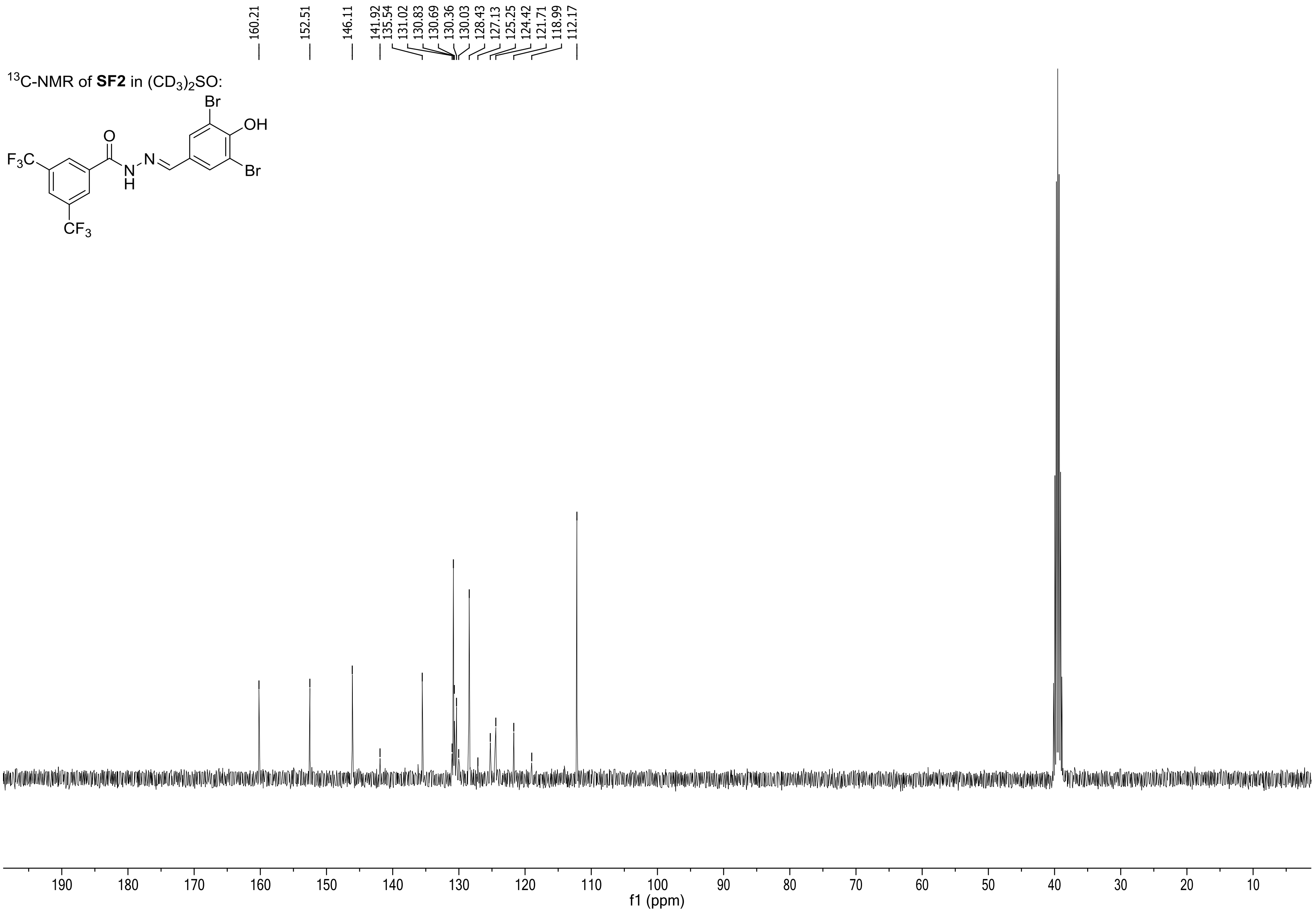

^{13}C-NMR of **SF2** in $(CD_3)_2SO$:
160.21
152.51
146.11
141.92
135.54
131.02
130.83
130.69
130.36
130.03
128.43
127.13
125.25
124.42
121.71
118.99
112.17
Br
OH
O
F_3C
N
N
H
Br
CF_3
190
180
170
160
150
140
130
120
110
100
90
80
70
60
50
40
30
20
10
f1 (ppm)

^{1}H-NMR of **SF3** in $(CD_3)_2SO$:

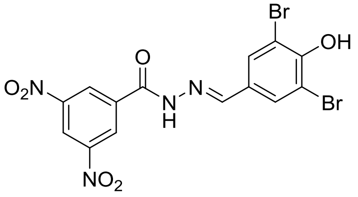

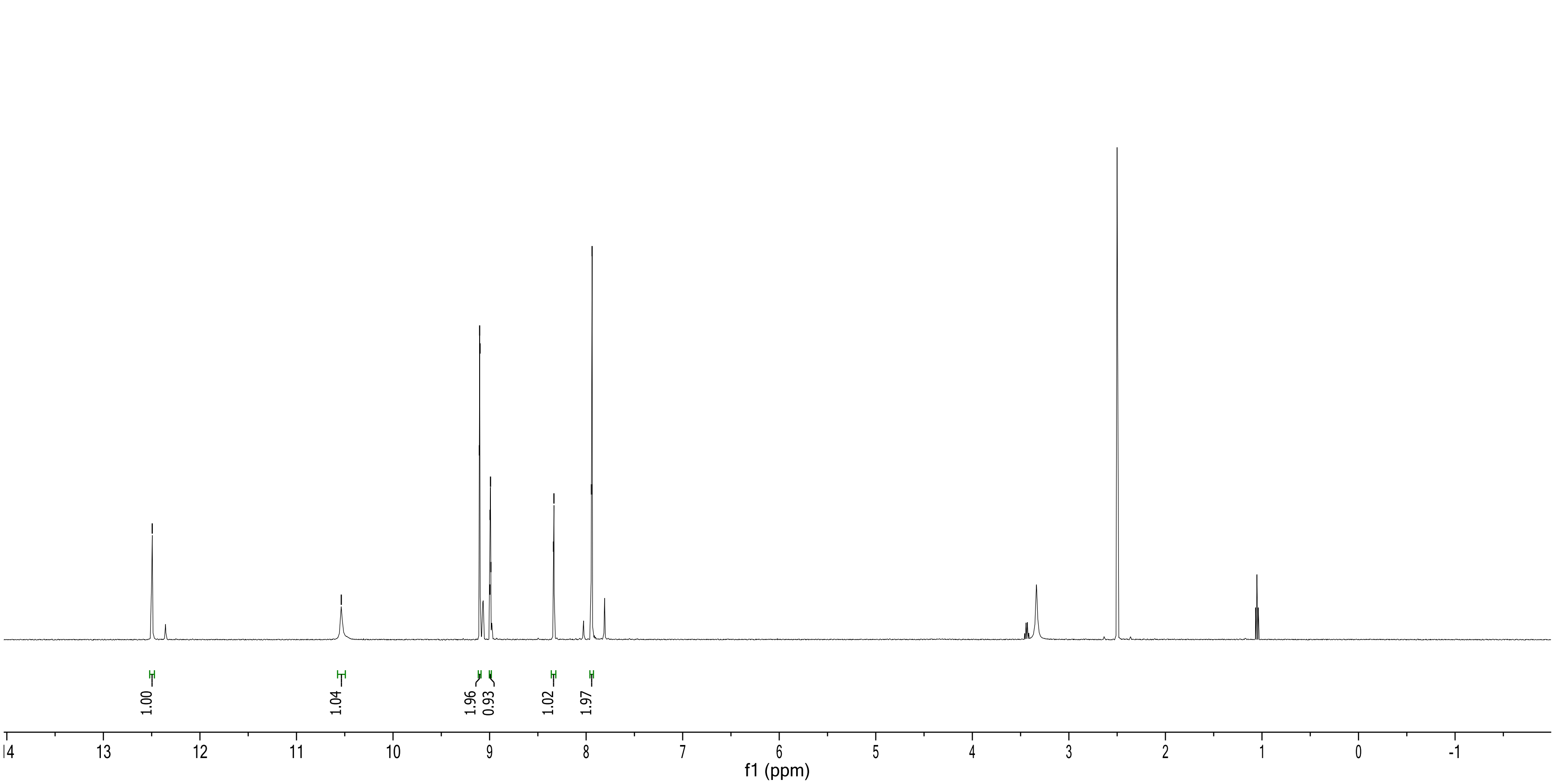

^{13}C-NMR of **SF3** in $(CD_3)_2SO$:

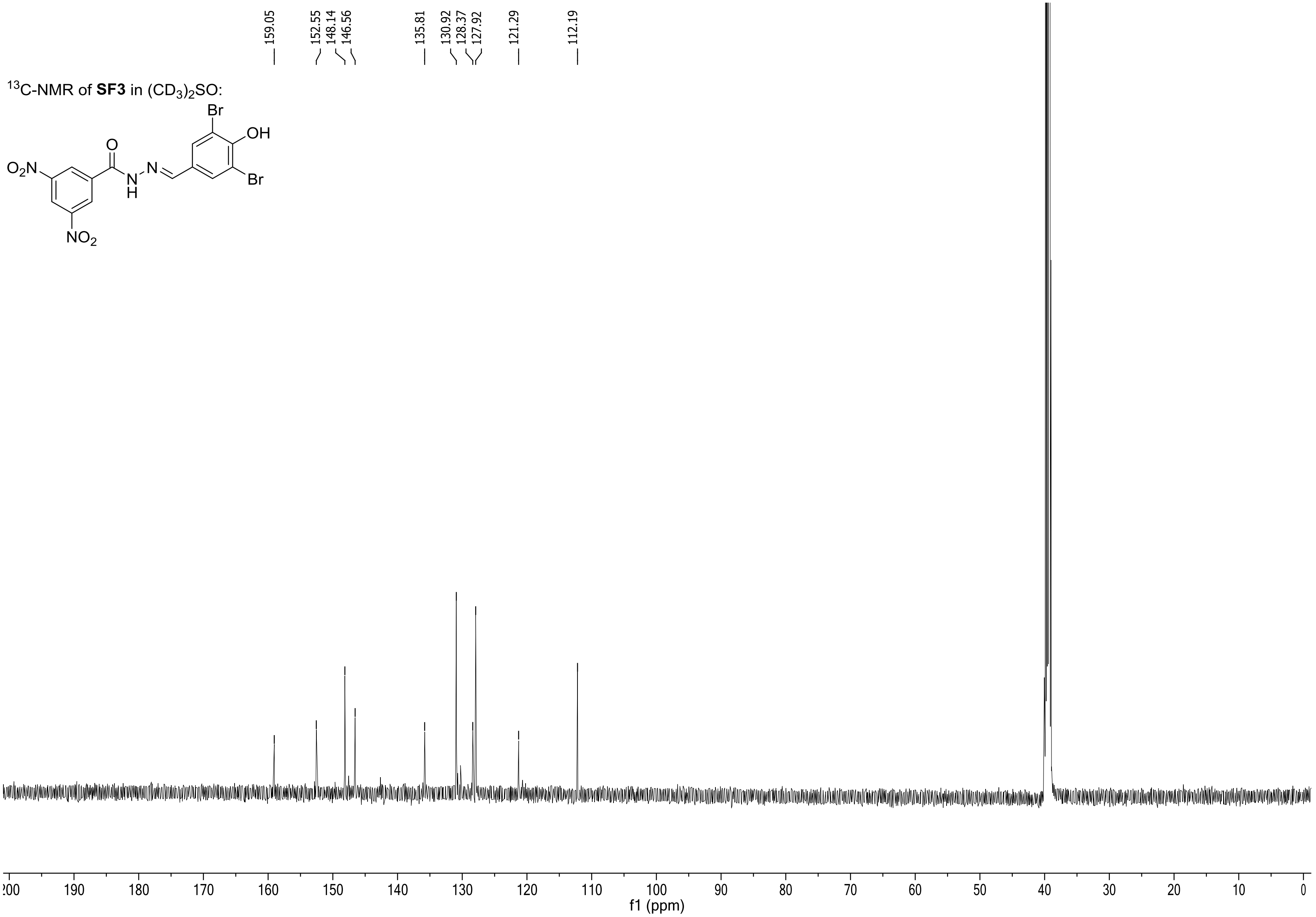

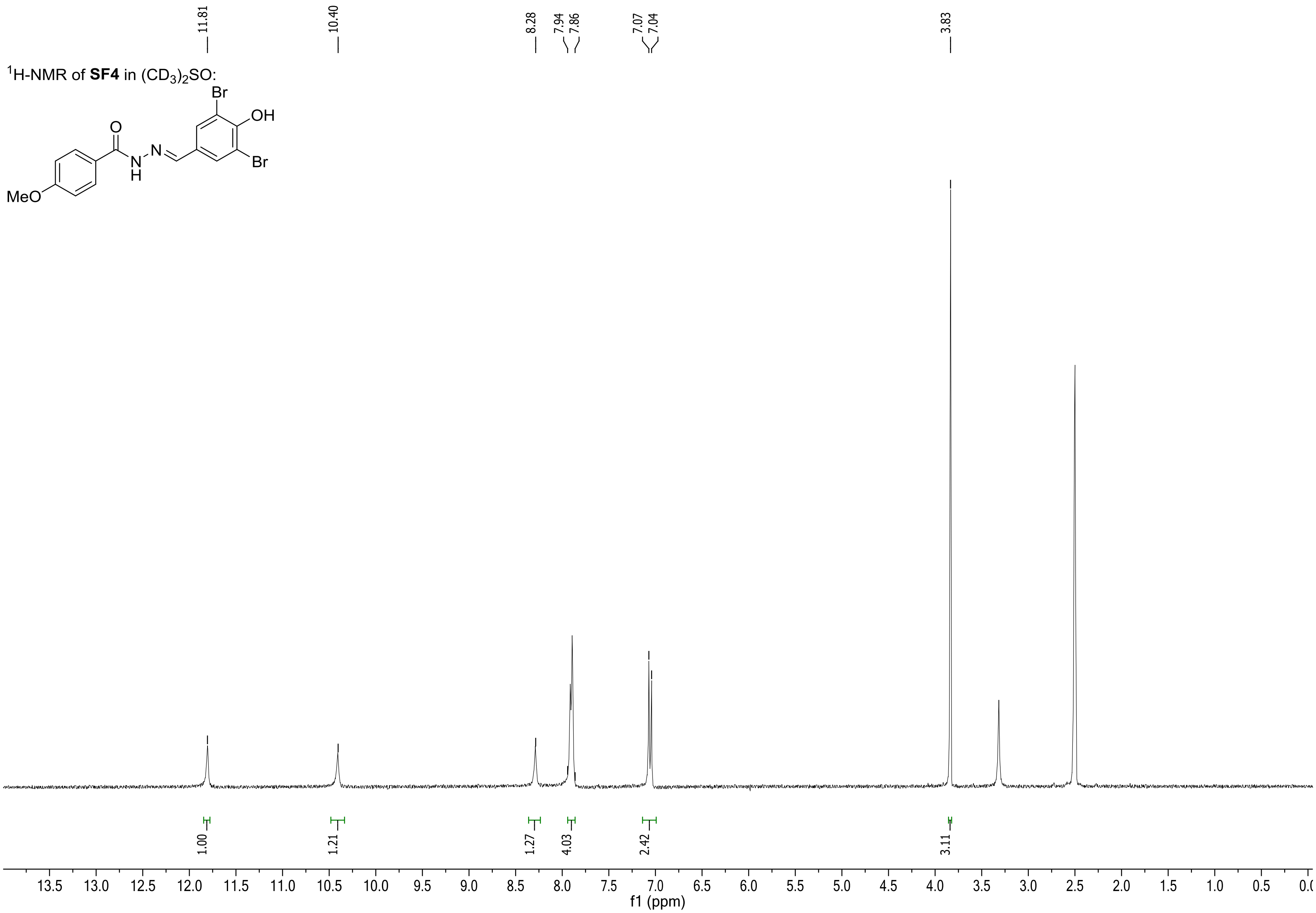
^{1}H-NMR of **SF4** in $(CD_3)_2SO$:
Br
OH
O
N
N
H
Br
MeO
11.81
10.40
8.28
7.94
7.86
7.07
7.04
3.83
1.00
1.21
1.27
4.03
2.42
3.11
13.5 13.0 12.5 12.0 11.5 11.0 10.5 10.0 9.5 9.0 8.5 8.0 7.5 7.0 6.5 6.0 5.5 5.0 4.5 4.0 3.5 3.0 2.5 2.0 1.5 1.0 0.5
f1 (ppm)

^{13}C-NMR of **SF4** in $(CD_3)_2SO$:

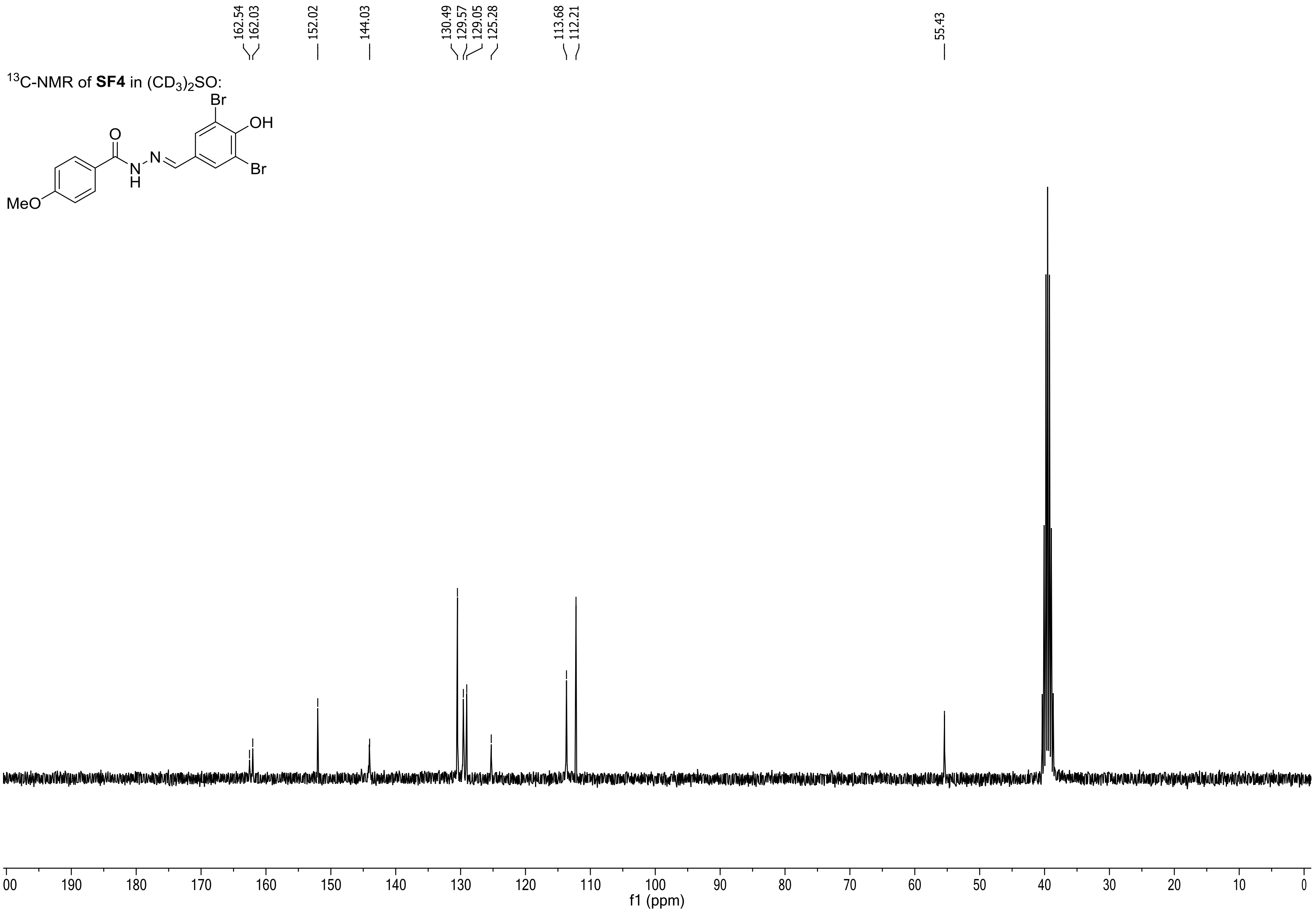

^{1}H-NMR of **SF5** in $(CD_3)_2SO$:

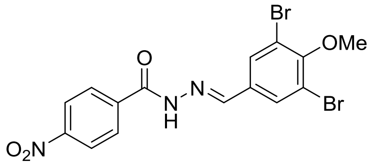

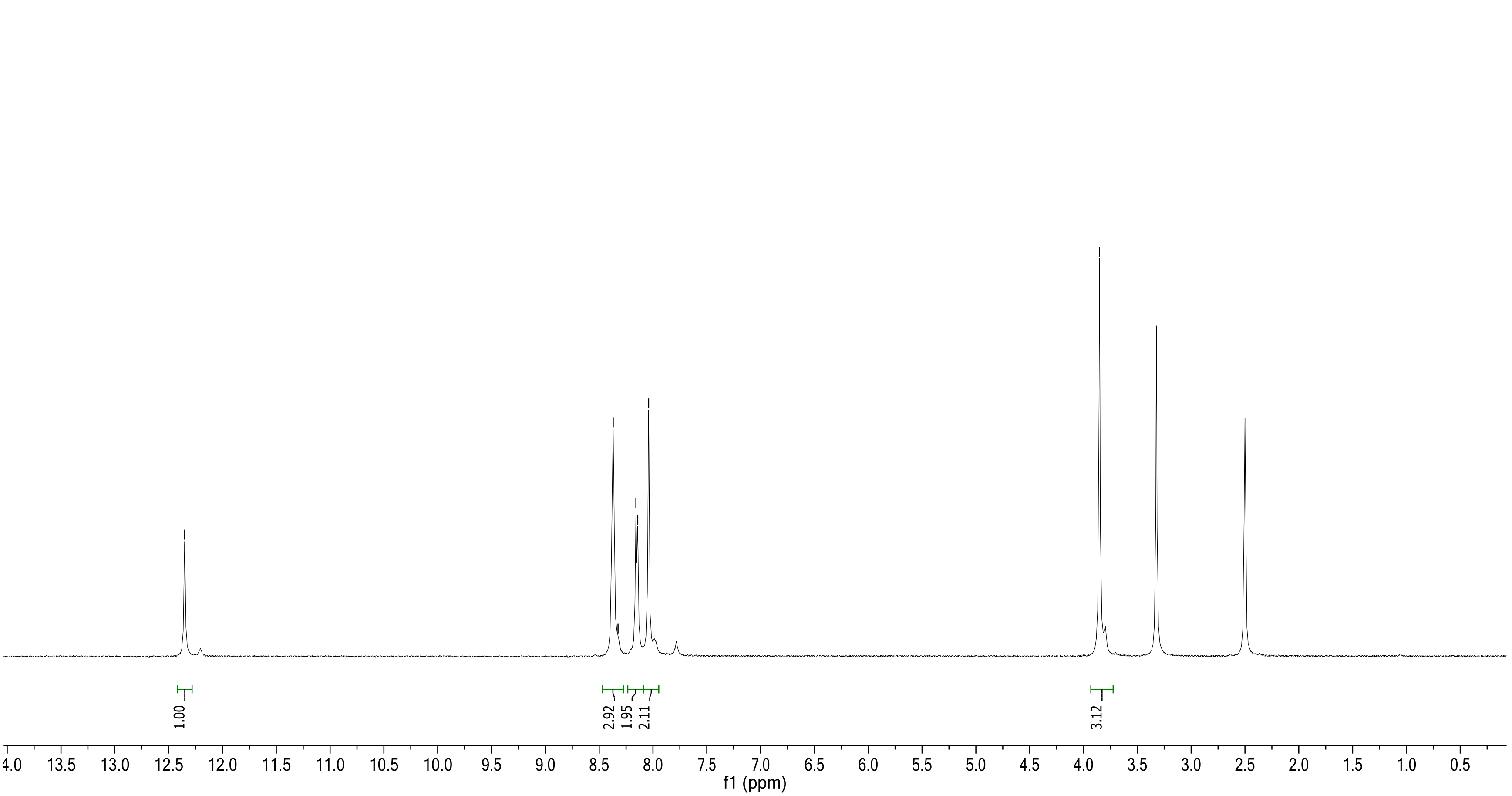

^{13}C-NMR of **SF5** in $(CD_3)_2SO$:

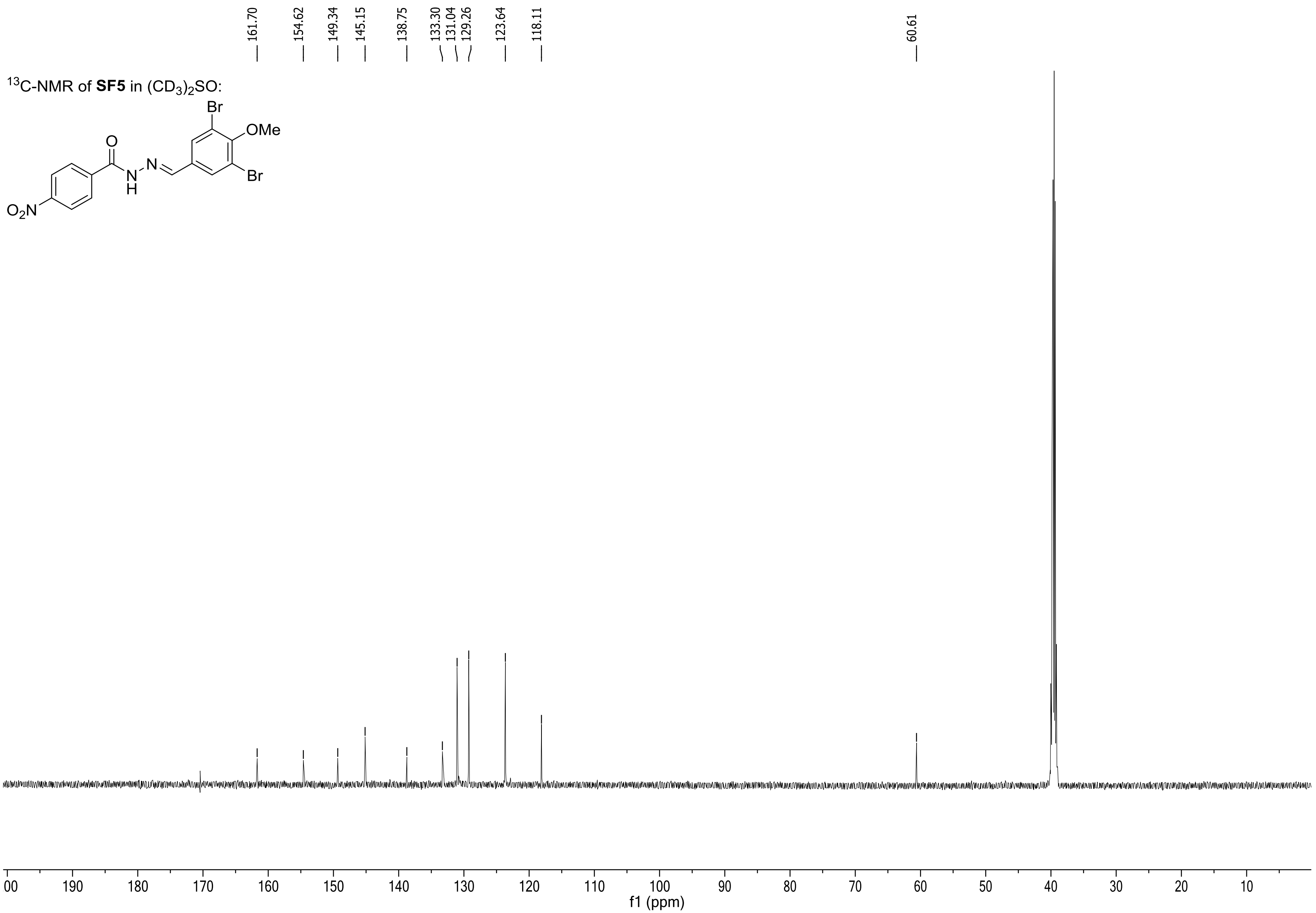

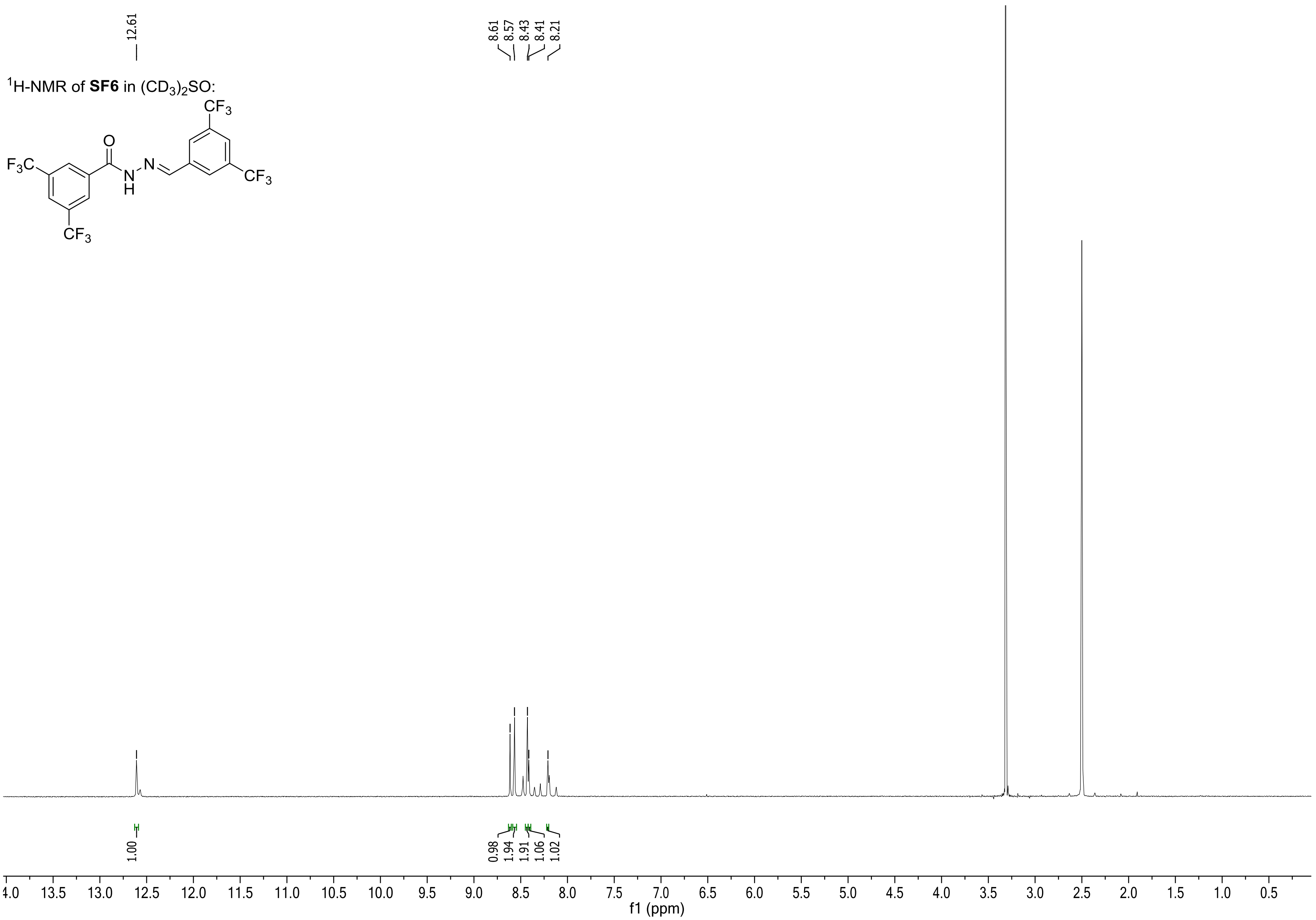

^{1}H-NMR of **SF6** in $(CD_3)_2SO$:
CF_3
O
F_3C
N
N
H
CF_3
CF_3
12.61
8.61
8.57
8.43
8.41
8.21
1.00
0.98
1.94
1.91
1.06
1.02
f1 (ppm)

^{1}H-NMR of **SF7** in $(CD_3)_2SO$:

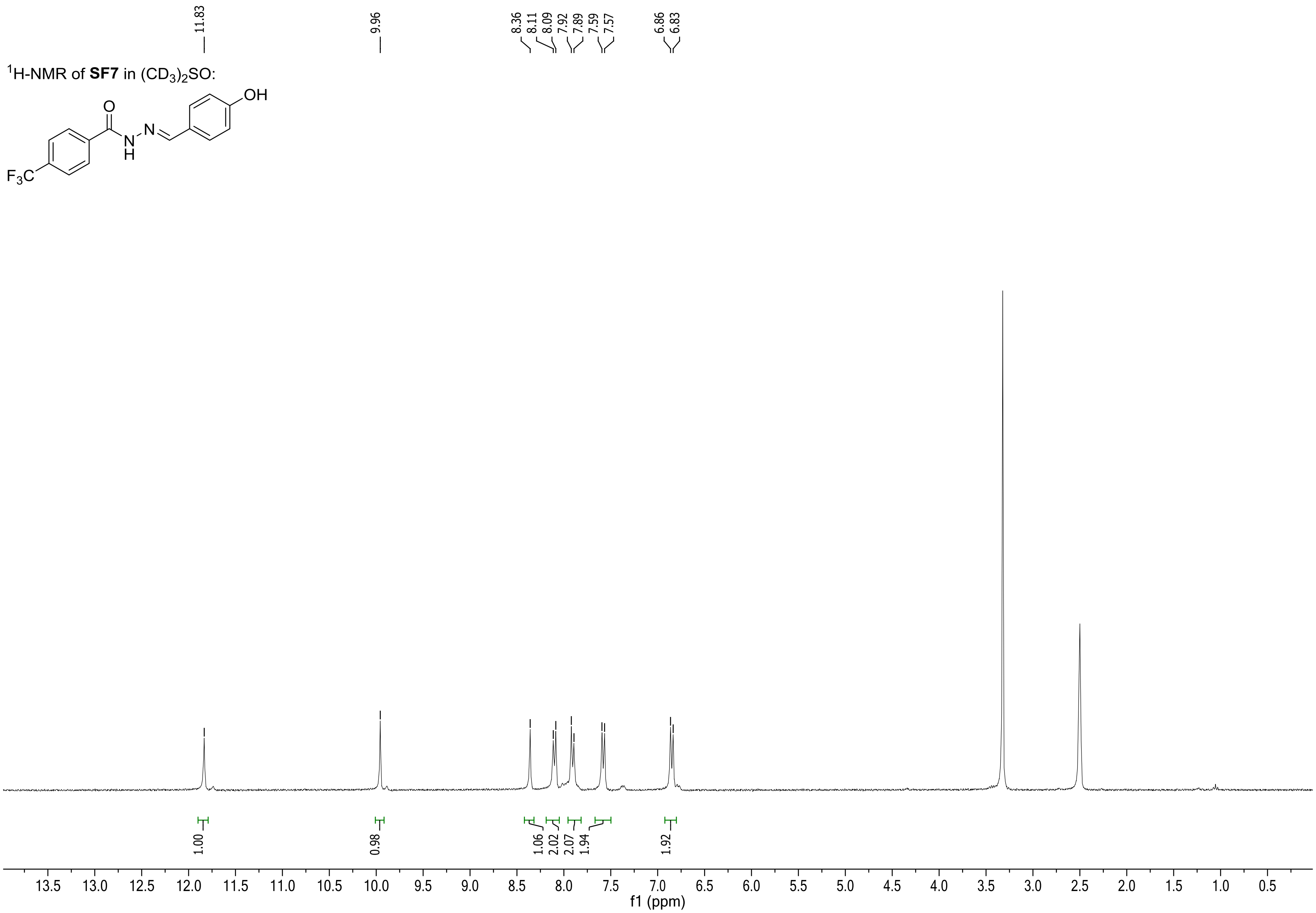

^{13}C-NMR of **SF7** in $(CD_3)_2SO$:

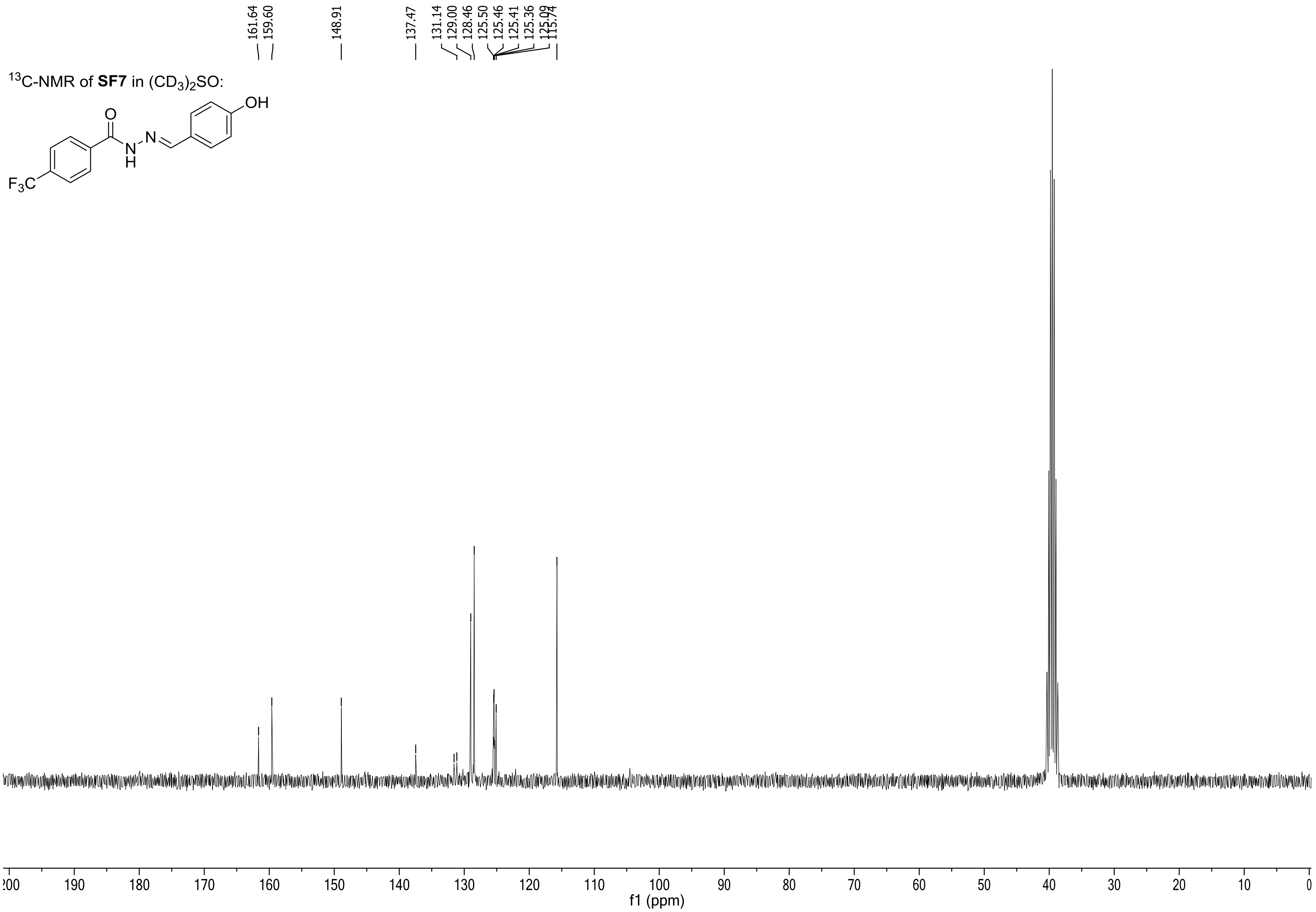

V. References

1. Karabanovich G, Zemanová J, Smutný T, Székely R, Šarkan M, Centárová I, Vocat A, Pávková I, Čonka P, Němeček J, Stolaříková J, Vejsová M, Vávrová K, Klimešová V, Hrabálek A, Pávek P, Cole ST, Mikušová K, Roh J. 2016. Development of 3,5-Dinitrobenzylsulfanyl-1,3,4-oxadiazoles and Thiadiazoles as Selective Antitubercular Agents Active Against Replicating and Nonreplicating Mycobacterium tuberculosis. J Med Chem 59:2362-80.
2. Inam A, Siddiqui SM, Macedo TS, Moreira DRM, Leite ACL, Soares MBP, Azam A. 2014. Design, synthesis and biological evaluation of 3-[4-(7-chloro-quinolin-4-yl)-piperazin-1-yl]-propionic acid hydrazones as antiprotozoal agents. Eur J Med Chem 75:67-76.
3. Wang Z, Zhang H, Killian BJ, Jabeen F, Pillai GG, Berman HM, Mathelier M, Sibble AJ, Yeung J, Zhou W, Steel PJ, Hall CD, Katritzky AR. 2015. Synthesis, Characterization and Energetic Properties of 1,3,4-Oxadiazoles. Eur J Org Chem 23:5183-88.
